# Efficient estimation of stereo thresholds: What slope should be assumed for the psychometric function?

**DOI:** 10.1371/journal.pone.0226822

**Published:** 2020-01-02

**Authors:** Ignacio Serrano-Pedraza, Kathleen Vancleef, William Herbert, Nicola Goodship, Maeve Woodhouse, Jenny C. A. Read

**Affiliations:** 1 Faculty of Psychology, Universidad Complutense de Madrid, Madrid, Spain; 2 Institute of Neuroscience, Newcastle University, Newcastle upon Tyne, United Kingdom; 3 Cognitive Neuropsychology Centre, Department of Experimental Psychology, University of Oxford, Oxford, United Kingdom; University of Wuerzburg, GERMANY

## Abstract

Bayesian staircases are widely used in psychophysics to estimate detection thresholds. Simulations have revealed the importance of the parameters selected for the assumed subject’s psychometric function in enabling thresholds to be estimated with small bias and high precision. One important parameter is the slope of the psychometric function, or equivalently its spread. This is often held fixed, rather than estimated for individual subjects, because much larger numbers of trials are required to estimate the spread as well as the threshold. However, if this fixed value is wrong, the threshold estimate can be biased. Here we determine the optimal slope to minimize bias and maximize precision when measuring stereoacuity with Bayesian staircases. We performed 2- and 4AFC disparity detection stereo experiments in order to measure the spread of the disparity psychometric function in human observers assuming a Logistic function. We found a wide range, between 0.03 and 3.5 log_10_ arcsec, with little change with age. We then ran simulations to examine the optimal spread using the empirical data. From our simulations and for three different experiments, we recommend selecting assumed spread values between the percentiles 60–80% of the population distribution of spreads (these percentiles can be extended to other type of thresholds). For stereo thresholds, we recommend a spread around the value σ = 1.7 log_10_ arcsec for 2AFC (slope *β* = 4.3 /log_10_ arcsec), and around σ = 1.5 log_10_ arcsec for 4AFC (*β* = 4.9 /log_10_ arcsec). Finally, we compared a Bayesian procedure (ZEST using the optimal σ) with five Bayesian procedures that are versions of ZEST-2D, Psi, and Psi-marginal. In general, for the conditions tested, ZEST optimal σ showed the lowest threshold bias and highest precision.

## Introduction

Bayesian and maximum-likelihood procedures are widely used in psychophysics to estimate detection thresholds. Given that these are parametric procedures, the experimenter has to provide in advance the parameters for the assumed psychometric function or model function [1]. Several simulation studies have shown the importance of choosing the adequate parameters. In general, when there is a mismatch between the subject’s psychometric function and the model function, threshold bias (i.e. the difference between the estimated and the real threshold) emerges [2–5]. Thus, before using Bayesian procedures (e.g. ZEST, [6]; QUEST, [7]), a previous experiment is ideally needed to estimate the subject’s psychometric function in order to avoid biased estimates. However, this will require more trials than is often practical, especially in a clinical setting. For this reason, parameters other than threshold are often held fixed at a constant value for all participants, with only the threshold fitted for each individual.

In most simulation studies that examine the mismatch between the subject’s psychometric function (*ψ*) and the assumed psychometric function (i.e. the model function, *M*), the authors use maximum-likelihood procedures, however, those results can be extrapolated to Bayesian procedures given that a Bayesian procedure using the mode of the posterior distribution as estimator and a uniform distribution as *a priori* distribution (instead of an informative distribution) is equivalent to the maximum likelihood estimation [1]. We therefore do not make a distinction between these approaches when discussing the literature.

One particularly important parameter of the psychometric function is the spread (σ), or equivalently, the slope of the model function. Slope and spread are inversely related; see S1 Appendix for details. Using a maximum-likelihood procedure, Green [4] (in a 2AFC task) found that the variability of the threshold estimate is lower when slope is underestimated, i.e. the model function was shallower (and its spread larger) than the observer’s true psychometric function. In line with this finding, Treutwein [1] recommends underestimating the slope (i.e. overestimating the spread or the standard deviation) relative to subject’s slope when using a dynamic stopping criterion. Emerson [2] found, using a maximum-likelihood procedure in a yes-no task, that threshold estimates were biased when the true slope was overestimated, whereas no bias was evident when the slope was underestimated. Madigan & Williams [3] (in a 2AFC task) compared Best PEST [8] and QUEST [7] using large lapse rates (20%). In general, they found that slope mismatches were not a serious problem for maximum-likelihood methods, however, they found that Best PEST procedure performs better (greater accuracy) when they *overestimate* the subject’s slope. Interestingly, they found the opposite for QUEST: underestimation of the true slope yielded better accuracy. Using Bayesian staircases (in a yes-no task) Alcalá-Quintana & García-Pérez [5] found that underestimating the subject’s slope (i.e. overestimating the spread) reduced the bias of the threshold estimation. This reduction was important if subject’s and assumed psychometric functions were different (e.g. Weibull vs. Logistic), however, they claimed no bias when both psychometric functions were identical. In summary, almost all studies recommend underestimating the subject’s slope (i.e. overestimating the spread) in order to get higher accuracy and smaller variance of the threshold estimates, but there are no recommendations about the degree of the underestimation needed to obtain thresholds with the smallest bias and standard deviation.

Our aim is to find the optimal slope (or spread) value in order to estimate stereo thresholds using a general Bayesian procedure. Our Bayesian procedure is described in the S1 Appendix (section A2). It assumes a uniform *a priori* distribution (non-informative prior); it uses the Logistic function for the model function; for selecting the stimulus intensity in each trial it computes the mean of the posterior distribution; and the threshold corresponds to the mean of the final distribution. This Bayesian configuration has the basic characteristics recommended from simulation studies [5, 6, 9, 10]. In summary, it is similar to ZEST [6] but using a uniform *a priori* distribution. Monte Carlo simulations confirm that with this procedure, better results are obtained when the slope is underestimated than when it is overestimated.

**Fig 1:**
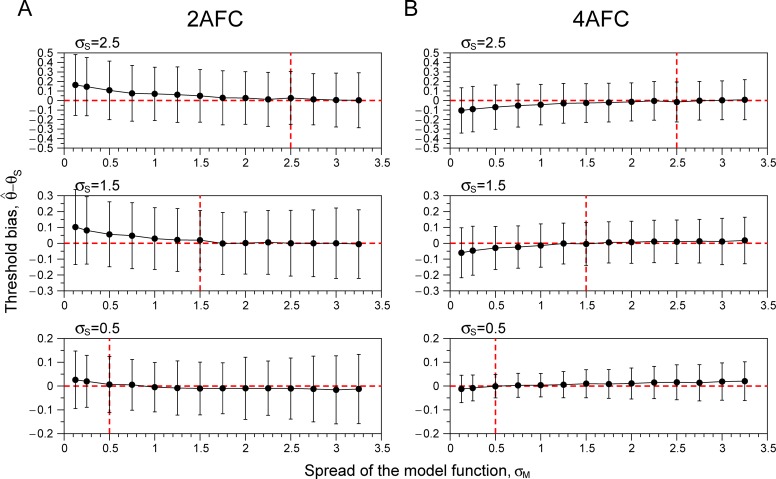
Simulations using adaptive Bayesian staircases. Each panel shows the mean threshold bias (estimated threshold-subject threshold) ± 1 SD, as a function of the spread (σ_M_) of the model function. The subject threshold was fixed to be θ_S_ = 1.5 (logarithmic value; 31.6 arcsec) that corresponds to the probability of correct responses of *π* = 0.75 for both tasks. Each panel shows the simulations for one particular spread of the subject (σ_S_ = [0.5, 1.5, 2.5]), the vertical red-dashed lines signals the spread of the subject psychometric function. The horizontal red-dashed line marks the zero-bias value. **A**. Results for a 2AFC task with guess rate γ_S_ = 0.5 and lapse rate *λ*_S_ = 0.02. **B**. Results for a 4AFC task with guess rate γ_S_ = 0.25 and lapse rate *λ*_S_ = 0.03. Lapse and guess rates were the same for the subject and the model function. Each dot corresponds to 2000 simulations of a Bayesian staircase of 30 trials (see S1 Appendix). Note that a spread value of the model function lower than the real one (subject’s spread) introduces bias in threshold estimation, positive bias in 2AFC and negative bias in 4AFC task (this is different if different probability of correct responses is chosen). See more details in the main text.

This is illustrated in Fig 1, which shows the simulation results for (A) 2AFC and (B) 4AFC tasks. Each panel shows the simulations for a different choice of the subject’s spread value (σ_S_). The abscissa values correspond to the spread value of the model function (σ_M_) used in the Bayesian procedure (larger spread values correspond to smaller slope values). Thus, the red dashed lines show the best case, where the assumed value is correct for the subject, σ_M_ = σ_S_. For the conditions tested, the results show a stronger bias for 2AFC (Fig 1A) than for 4AFC (Fig 1B). In general, all panels show the classical result, overestimation of the subject’s slope (smaller spread than the subject’s spread) produces stronger bias than underestimation of the subject’s slope (or overestimation of the spread). The vertical dashed red line signals the spread value of the subject. For both tasks and for almost all spread values of the subject, bias and SD rise more slowly to the right of this line than to the left. Thus, in agreement with previous results, underestimation of the slope (overestimation of the spread values) yields better results than overestimation [4].

However, Fig 1 also shows that thresholds become more biased and less reliable if the slope underestimation is too severe (i.e. σ_M_ is too high). Thus, there is no unique model spread value (σ_M_) that works well for all possible spreads of any observer (σ_S_). However, it would be possible to find the optimal spread value if the distribution of spreads for the particular task is known. We could then use Monte Carlo simulations to estimate the optimal model spread (σ_M_) that minimizes bias and maximizes precision for the given distribution of spreads in the population.

It is well known that there are Bayesian methods like MUEST [11], ZEST 2D [12], Psi [13], Psi-marginal [14] or QUEST+ [15] that can be used to estimate the threshold and the slope simultaneously (and some of them the lapse rate too), however this has a cost in terms of number trials needed or for example, when the slope parameter is unknown, MUEST (a multidimensional extension of QUEST to estimate threshold and the slope simultaneously) performs better than QUEST [7], on the other hand, if the slope parameter is known, QUEST performs better (i.e. lower threshold bias) than MUEST [11]. There are versions of ZEST 2D, Psi-marginal, or QUEST+ that can be modified to estimate only the threshold parameter treating the slope parameter as a nuisance parameter or marginalizing it, however, it is not known whether they will perform better than a well-known Bayesian method like ZEST with the optimal slope.

Thus, in this paper, our objectives are: (i) to determine the optimal slope value for the model function in order to estimate reliable thresholds on stereoscopic disparity detection tasks when using Bayesian procedures; (ii) to quantify the threshold bias and imprecision expected for subjects with different values of spread. To this end, we have performed three different stereo experiments (two measuring global stereopsis and one measuring local stereopsis) in which we estimated the threshold, the spread and lapse rate of 260 participants using an adaptive weighted one-up one-down staircase in two tasks, 2AFC and 4AFC. We have performed Monte Carlo simulations using the distributions of the empirical spreads from those three experiments and we have estimated an optimal spread value for each experiment. (iii) Finally, in order to test the effectivity of using the optimal slope in a Bayesian procedure, we will compare a Bayesian method (ZEST with optimal σ) with other five Bayesian procedures performing Monte Carlo simulations using the empirical data (thresholds, slopes, and lapse rates) of each subject as model psychometric function and two tasks (2AFC and 4AFC).

## Materials and methods

### Participants

We performed three experiments with different number of participants. In Experiment 1 (2AFC_global) we tested 79 participants (33 females) (aged from 4.6 to 61 years, mean = 19.5 years, SD = 14.05). In Experiment 2 (4AFC_global) we tested 82 participants (37 females) (aged from 5 to 21.8 years, mean = 10.02 years, SD = 3.14). In Experiment 3 (4AFC_local) we tested 99 participants (45 females) (aged from 4.75 to 36.08 years, mean = 10.09 years, SD = 4.86). All subjects completed 80 trials or more up to 120 trials. All subjects decided at the beginning of the experiment whether perform the experiment with 80 or with 120 trials. Adults and children were recruited at the Centre for Life science center in Newcastle upon Tyne, UK (http://www.life.org.uk/). Adult participants provided informed written consent. The parents or other accompanying adults of child participants provided written consent for children. The study protocol was compliant with the Declaration of Helsinki and was approved by the Ethics Committee of the Newcastle University Faculty of Medical Sciences (approval number 00625).

### Equipment

**Fig 2:**
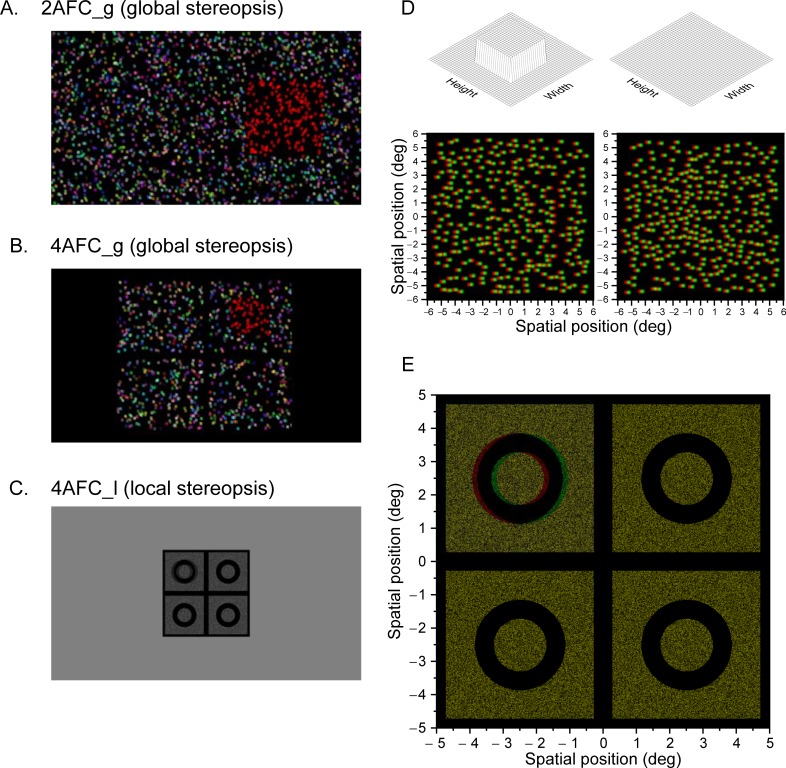
Screen capture examples of the stimuli used in the experiments. **A**. Example of the stimulus presented in Experiment 1 (2AFC_global). This capture corresponds to a practice trial where in addition to the disparity the target area was also presented in red (this colour/luminance cue was removed after the practice trials). **B**. Example of the stimulus used in Experiment 2 (4AFC_global). **C**. Example of the stimulus used in Experiment 3 (4AFC_local). **D**. Anaglyph version of the stimulus used in Experiments 1 and 2. **E**. Anaglyph version of the stimulus used in Experiment 3. To observe correctly these examples with anaglyph glasses, place the red filter in front of the left eye.

Stimuli were presented on a 23-inch passive 3D monitor (D2367PH, AOC) that uses passive polarization with a spatial resolution of 1920 × 1080 pixels (52 cm × 29 cm) and a refresh rate of 60 Hz. Left and right images were separated by circular-polarization 3D glasses. Observers were seated at 90 cm from the monitor, so a pixel subtended 60.4 arcsec. To stabilize the observer’s head and to control the observation distance a chin rest (UHCOTech HeadSpot, Houston, TX) was used. In the 2AFC task (Experiment 1), subjects indicated their response by pressing the left or right button of a standard computer mouse. In the 4AFC tasks Experiment 2 & 3), subjects responded via a 4-button response box (ResponsePixx HandHeld, VPixx Technologies). The four corner buttons corresponded with the four spatial locations of the 4AFC (see Fig 2B). Data were collected on a DELL workstation (Intel(R) Core (TM) i3 CPU 540 @3.07GHz, 4GB RAM, 64- bit Operating System, Windows 7), with a GeForce GTX 460 graphics card (NVIDIA), running MATLAB R2012a, 64-bit (Mathworks). The experiments were programmed using Psychophysics Toolbox extensions [16–18].

### Stimuli

All stimuli were generated using Matlab (R2012a, Mathworks). In Experiments 1 (2AFC_global) and 2 (4AFC_global), we designed the stimuli to measure global (or cyclopean) stereopsis. These experiments differed only in the number of alternatives. In Experiment 3 (4AFC_local), using a 4AFC task, we measured local (feature or contour-based) stereopsis. The stimulus used is a version of the Randot Circles stereotest (Stereo Optical Company, Inc., USA) commonly used in clinical screening.

For Experiments 1 and 2, we used dynamic random dots of different colors on a black background (see Fig 2A and 2B). The color of the dots was generated by selecting the RGB values independently from a uniform distribution between minimum and maximum luminance. The position and color of the dots was random and updated every frame at 60 Hz. The dots were generated using the function “Screen(‘DrawDots’)” of the Psychophysics Toolbox extensions [16–18]. Given that the stereoscopic presentation system uses line interleaving to dissociate left and right images, the dots appeared as ellipses, with a width of 10 pixels and a height of 20 physical pixels (10.07 × 20.14 arcmin). Subpixel disparities were achieved by using an antialiasing technique (see [19]). In Experiment 1, we used a 2AFC task and the target was a random dot stereogram of 8.4 × 8.4 deg with crossed disparities presented on top of a surround composed of random dots with uncrossed disparities (see Fig 2A). The target was presented on the left or right side of the screen at 9.3 degrees from the midline. In Experiment 2, we used a 4AFC task, and the target was also a random dot stereogram of 4.3 × 4.3 deg with crossed disparities presented in one of the four corners of the screen 6.5 deg away from the centre (see Fig 2B). In this experiment the target was located in the center a surrounding rectangle of random dots with uncrossed disparities (9.3 × 7.4 deg, width × height). Target and background had equal and opposite disparity relative to the screen. This procedure reduces monocular cues that could be present for high disparities [19]. The stimulus disparity was defined as the relative disparity between the target and background (see an anaglyph version in Fig 2D).

Fig 2C shows an example of the stimulus used in Experiment 3. The stimulus consisted of four black circles on a square background filled with static white noise. Each square had a size of 5 deg and each circle a diameter of 2.65 deg. The circle was constructed using a 2D isotropic window with a Butterworth profile (see equation in Appendix A in [20]), with this luminance profile we can achieve subpixel disparities. We applied the disparity to one of the circles and the background was set to zero disparity (see anaglyph version in Fig 2D).

### Procedure

**Fig 3:**
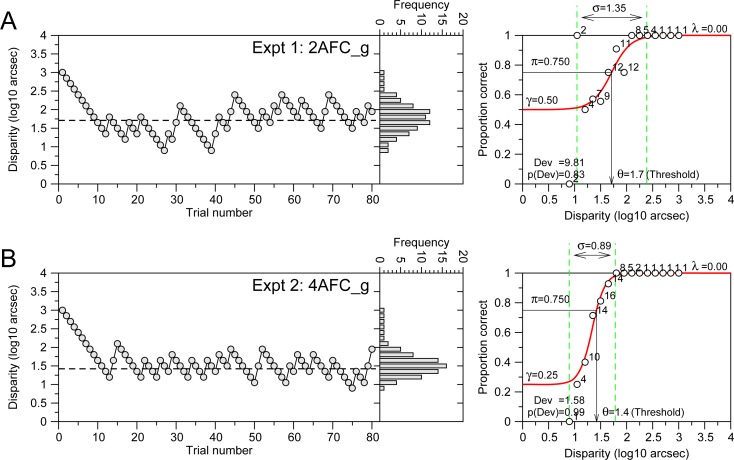
Example of staircases used in the experiments. Left panels, staircases and histograms of disparities presented. Right panels show the fitting of the psychometric function (red line) to the data (white dots). The number on the right of white dots corresponds to number of times that a disparity value was presented. **A**. Example of one participant (22.9 years) performing Experiment 1 (2AFC_g). **B**. Example of one participant (11.4 years) performing Experiment 2 (4AFC_g).

In all experiments we used the same adaptive weighted one-up one-down staircase (see a simulation study about this type of staircase in [21]). The staircase started with a practice trial at a disparity of 3 log_10_ arcsec (or 1000 arcsec). In the first trial of Experiments 1 and 2, in addition to the disparity, all target dots were presented in red at maximum luminance (see Fig 2A and 2B). This non-stereo color/luminance cue was added to the practice trial to ease understanding of the task. This cue was eliminated after the first trial; therefore, after the first trial the target could only be discriminated based on disparity. The staircase works in log_10_ values of the disparity. After each correct response, log-disparity was reduced by subtracting 0.15 log_10_ arcsec (i.e. disparity was divided by a factor of 1.41). Following each incorrect answer, log-disparity was increased by three times this value or 0.45 log_10_ arcsec (i.e. disparity was increased by a factor of 2.81; see two examples of this staircase in Fig 3, left panels). In total, 94% of the participants completed 80 trials and 6% 120 trials. No feedback about correctness of responses was provided. The stimulus on each trial was presented until the participants made a response, thus the experiment proceeded at a pace determined by the participant. All experiments were carried out in a dimmed area at the Centre for Life.

### Fitting procedure

First, we computed the proportion of correct responses observed for each disparity presented to the subject. Then, for each subject we fitted the Logistic psychometric function (described in S1 Appendix; red lines on the right panels of Fig 3) to the proportion of correct responses by the method of maximum likelihood, using the function “fminsearch” of Matlab. This is the same function that will be used as a model function in the Bayesian staircases. We fitted the psychometric function with three free parameters, threshold, θ; spread, σ; and lapse rate, λ, with constrains θ>0, σ>0, and 0<λ<0.06. The guess parameter γ was fixed to 0.5 for 2AFC and 0.25 for 4AFC. We established the disparity threshold as the value that corresponds to the probability of correct responses of π = 0.75 for both, 2AFC and 4AFC tasks. We assessed the goodness of fit of the psychometric function using the likelihood ratio or *deviance* (Dev) described in equation 5 of Wichmann & Hill [22]. We fixed a priori the ranges for valid fittings, θ ϵ[1, 500] arcsec; σ ϵ[0.01, 7]; and the probability p(Dev) associated to the *deviance* p(Dev)>0.05. Those subjects that didn’t meet these criteria were discarded from the analysis. The lower value of the range for σ (0.01) was chosen taking into account that previous simulation studies, with this type of adaptive staircase, have shown spread estimation errors (spread values close to 0) for a small number of trials (see Fig 4 of [21]).

## Results

**Fig 4:**
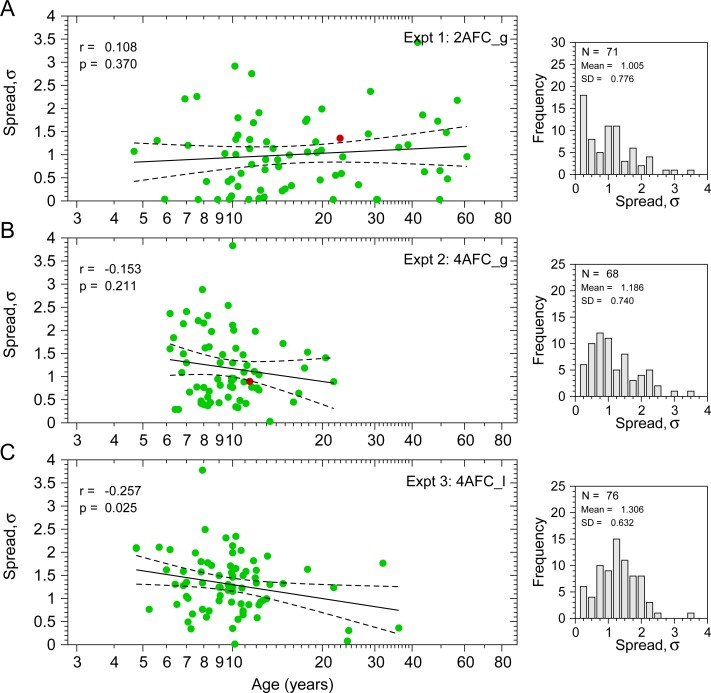
Spread values from Experiments 1 (2AFC_g), 2 (4AFC_g), & 3 (4AFC_l). Left panels show the spread value (σ) (i.e. inverse of the slope, with units of log_10_ arcsec) as a function of age. Right panels show the distribution of spreads for each experiment. **A**. Results from Experiment 1. **B**. Experiment 2; **C**. Experiment 3. Green dots are the individual spreads obtained from the fitting. The red dots in panels A and B correspond to the spread values of the participants described in Fig 3. Black lines are fitted regression lines (dashed lines: 95% regression confidence interval for the mean). **A**. $\hat{\sigma }$(age)_2AFC_g_ = 0.632+0.308×log_10_(age). **B**. $\hat{\sigma }$(age)_4AFC_g_ = 2.097–0.924×log_10_(age). **C**. $\hat{\sigma }$(age)_4AFC_l_ = 2.295–0.996×log_10_(age). Top left of left panels shows the Pearson correlation between age and thresholds (log_10_(age) and σ).

Fig 4 shows the spread (σ) of the participants’ psychometric function as a function of age for three experiments. Green dots show the spread value obtained from the fitting procedure. The averages of the spreads (σ) for each Experiment 1 were, in log_10_ arcsec: $\bar{\sigma }$_2AFC_g_ = 1.005 ± 0.776 (mean ± SD); $\bar{\sigma }$_4AFC_g_ = 1.186 ± 0.74; $\bar{\sigma }$_4AFC_l_ = 1.306 ± 0.632. A spread of 1 log_10_ arcsec means that 95% of the range of the psychometric function occurs over a factor of 10 in disparity. For example, if performance is 2.5% above chance when disparity is 10 arcsec, then with σ = 1 log_10_ arcsec it will be 2.5% below maximum when disparity is 100 arcsec. Conversely, a spread of 1.3 log_10_ arcsec corresponds to a factor of 20.

In order to compare the spreads of the three experiments we performed the Levene’s test for homogeneity of variances (F(2,212) = 1.872, p = 0.156), and the Shapiro-Wilk normality test showing that only the distribution of spreads in Experiment 3 showed normality (p>0.05). Thus, we have performed two tests, a parametric one-way analysis of variance (ANOVA) and a non-parametric Kruskal-Wallis test. Both tests produce the same conclusions. ANOVA shows significant differences between σ estimates from the three experiments (F(2,212) = 3.267, p = 0.04;). Post-hoc tests for multiple comparisons using Bonferroni correction only shows significant differences between Experiment 1 (2AFC_g) and Experiment 3 (4AFC_l) (p = 0.035). Kruskal-Wallis test was conducted to compare the three distributions of spreads and results shows again significant differences (*χ*^2^ = 8.462,*p* = 0.015,*d*.*f*. = 2). Pairwise comparisons show significant differences between Experiment 1 and 3 (p = 0.011) too.

Correlation analysis shows no significant correlation in Experiment 1 between spread (σ) and age (log_10_(age)) (r = 0.108, p = 0.370, N = 71); and also, in Experiment 2 (r = -0.153, p = 0.211, N = 68). Experiment 3 shows a significant negative correlation (r = -0.257, p = 0.025, N = 76). However, after computing the Cook’s distance in order to detect and remove highly influential observations (observations with Cook’s distance higher than three times the mean of all Cook’s distances), no significant correlations are found (r_2AFC_g_ = 0.196, p = 0.1209, N = 64; r_4AFC_g_ = -0.153, p = 0.235, N = 62; r_4AFC_l_ = -0.159, p = 0.1887, N = 70). Therefore, our results suggest that spread values are independent of age.

**Fig 5:**
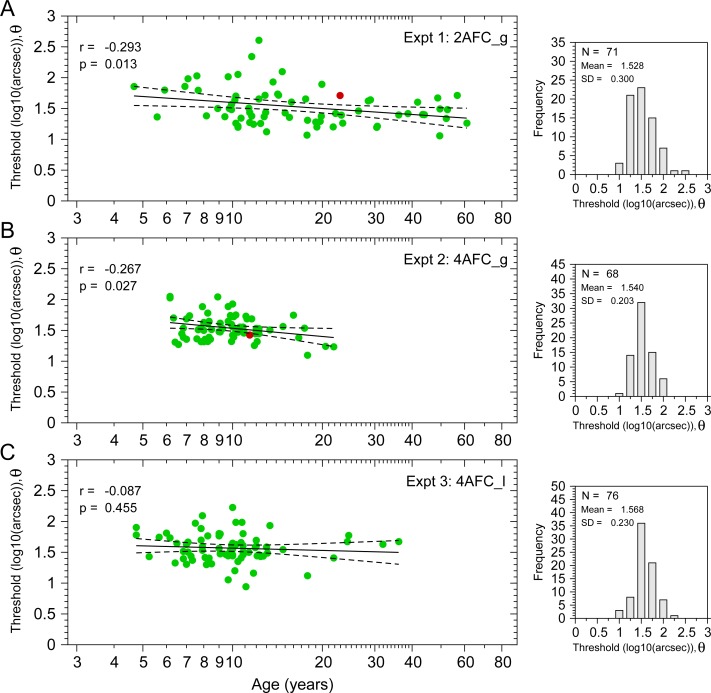
Disparity thresholds from Experiments 1 (2AFC_g), 2 (4AFC_g), & 3 (4AFC_l). Left panels show the stereoacuity thresholds (log_10_(arcsec)) as a function of age. Right panels show the distribution of thresholds for each experiment. **A**. Results from Experiment 1. **B**. Experiment 2; **C**. Experiment 3. Green dots are the individual thresholds obtained from the fitting that correspond to a probability of correct response of 0.75. The red dots in panels A and B correspond to the thresholds of the participants described in Fig 3. Black lines are fitted regression lines (dashed lines: 95% regression confidence interval for the mean). **A**. $\hat{\theta }$(age)_2AFC_g_ = 1.919–0.323×log_10_(age). **B**. $\hat{\theta }$(age)_4AFC_g_ = 1.974–0.441×log_10_(age). **C**. $\hat{\theta }$(age)_4AFC_l_ = 1.69–0.123×log_10_(age). Top left of left panels shows the Pearson correlation between age and thresholds (log_10_(age) and log_10_(arcsec)).

Fig 5 shows the disparity thresholds (θ, in log_10_ units) as a function of age from the three experiments. Green dots show the disparity threshold obtained from the fitting procedure. The averages of the thresholds (in log_10_(arcsec)) for each Experiment were: $\bar{\theta }$_2AFC_g_ = 1.528 ± 0.3 (mean ± SD) (33.73 arcsec, N = 71); $\bar{\theta }$_4AFC_g_ = 1.54 ± 0.203 (34.67 arcsec, N = 68); $\bar{\theta }$_4AFC_l_ = 1.568 ± 0.23 (36.98 arcsec, N = 76).

Multiple studies have found that stereoacuity improves (i.e. disparity thresholds decrease) with age until the age of 10 years [23–27], remains steady until the age of 50–60 years and then stereoacuity decreases [27–29]. Given that our youngest participants are around 5 years and very few are over 50 (see Fig 5), we might expect to see a negative correlation between age and disparity thresholds. Pearson’s product-moment correlation between age (log_10_(age)) and thresholds (log_10_(arcsec)) did indeed show a significant negative correlation (r_2AFC_g_ = -0.293, p = 0.013, N = 71) for Experiment 1 and for Experiment 2 (r_4AFC_g_ = -0.267, p = 0.027, N = 68). However, for Experiment 3, we found no correlation (r_4AFC_l_ = -0.087, p = 0.455, N = 76). We also computed the Cook’s distance in order to detect highly influential observations. Only Experiment 1 still shows significant correlations after removing highly influential observations (r_2AFC_g_ = -0.32, p = 0.008, N = 66; r_4AFC_g_ = -0.146, p = 0.261, N = 61; r_4AFC_l_ = -0.052, p = 0.668, N = 69). The absence of correlation in the 4AFC experiments is presumably related to the lower age-range of the participants.

**Fig 6:**
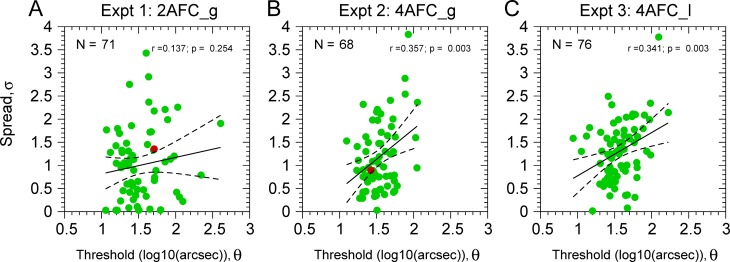
Results from Experiments 1 (2AFC_g), 2 (4AFC_g), & 3 (4AFC_l). Each show the spread value (σ) (i.e. inverse of the slope, in log_10_ arcsec) as a function of the disparity threshold (θ, in log_10_(arcsec)). **A**. Results from Experiment 1. **B**. Experiment 2; **C**. Experiment 3. Green dots are the individual spreads and thresholds obtained from the fitting. The red dots in panels A and B correspond to the spread and thresholds values of the participants described in Fig 3. Black lines are fitted regression lines (dashed lines: 95% regression confidence interval for the mean). **A**. $\hat{\sigma }$(θ)_2AFC_g_ = 0.462+0.355×θ **B**. $\hat{\sigma }$(θ)_4AFC_g_ = -0.823+1.305×θ. **C**. $\hat{\sigma }$(θ)_4AFC_l_ = -0.159+0.934×θ. Top right of the panels shows the Pearson correlation between thresholds and spread (θ and σ).

Fig 6 shows the spreads (σ) as a function of the thresholds (θ) for all participants. Correlation analysis shows no correlation for Experiment 1 (r = 0.137, p = 0.254, N = 71) and significant correlations for Experiment 2 (r = 0.357, p = 0.003, N = 68) and Experiment 3 (r = 0.341, p = 0.003, N = 76). After removing influential observations using Cook’s distance, the correlations barely changed (r_2AFC_g_ = 0.1, p = 0.428, N = 64; r_4AFC_g_ = 0.349, p = 0.0049, N = 63; r_4AFC_l_ = 0.2906, p = 0.0133, N = 72). Therefore, although the correlations are not very strong, 4AFC data shows that the lower the thresholds the steeper the psychometric function (small spread).

Finally, we analyzed the estimated lapse rate. We define the “lapse rate” parameter λ to be the probability of responding incorrectly as the result of a lapse in attention etc. The average values fitted for the three experiments were $\bar{\lambda }$_2AFC_g_ = 0.0103 ± 0.021 (mean ± SD) (N = 71); $\bar{\lambda }$_4AFC_g_ = 0.0231 ± 0.0271 (N = 68); $\bar{\lambda }$_4AFC_l_ = 0.0208 ± 0.027 (N = 76). Levene’s test for homogeneity of variances shows heteroscedasticity (F(2,212) = 16.366, p<0.001) and Shapiro-Wilk normality test shows that the three distributions of lapse rates are not normally distributed. Although the distributions show heteroscedasticity, we performed a non-parametric Kruskal-Wallis test that shows significant differences between the distributions of lapse rates (*χ*^2^ = 9.784,*p* = 0.008,*d*.*f*. = 2). Pairwise comparisons show significant differences only between lapse rates of Experiment 1(2AFC_g) and Experiment 2 (4AFC_g) and almost significant (p = 0.058) between Experiment 1 and 3(4AFC_l) (Welch’s ANOVA show the same results, F(2,138.868) = 5.943, p = 0.003, and significant differences between Experiment 1 and 2 (p = 0.01) and between 1 and 3 (p = 0.04)). This is not a surprising result given that with the same probability of making a lapse (λ*), the probability of responding incorrectly as a result of a lapse increases with the number of alternatives [30]: *λ* = λ*(1−*γ*). Our data indicate the following estimates for the probability of making a lapse: $\bar{\lambda^{*}}$_2AFC_g_ = 0.0207 ± 0.042 (mean ± SD) (N = 71); $\bar{\lambda^{*}}$_4AFC_g_ = 0.0308 ± 0.0361 (N = 68); $\bar{\lambda^{*}}$_4AFC_l_ = 0.0276 ± 0.036 (N = 76). Shapiro-Wilk test shows confirms that the distributions are not normally distributed, however, Levene’s test shows homogeneity of variance (F(2,212) = 0.072, p<0.930). Kruskal-Wallis test shows no significant differences between the distributions of lapse rates (*χ*^2^ = 4.686,*p* = 0.096,*d*.*f*. = 2). Thus, the probability of making a lapse is similar in the three Experiments (0.02–0.03, a percentage of 2–3%).

## Determination of the optimal slope value

**Fig 7:**
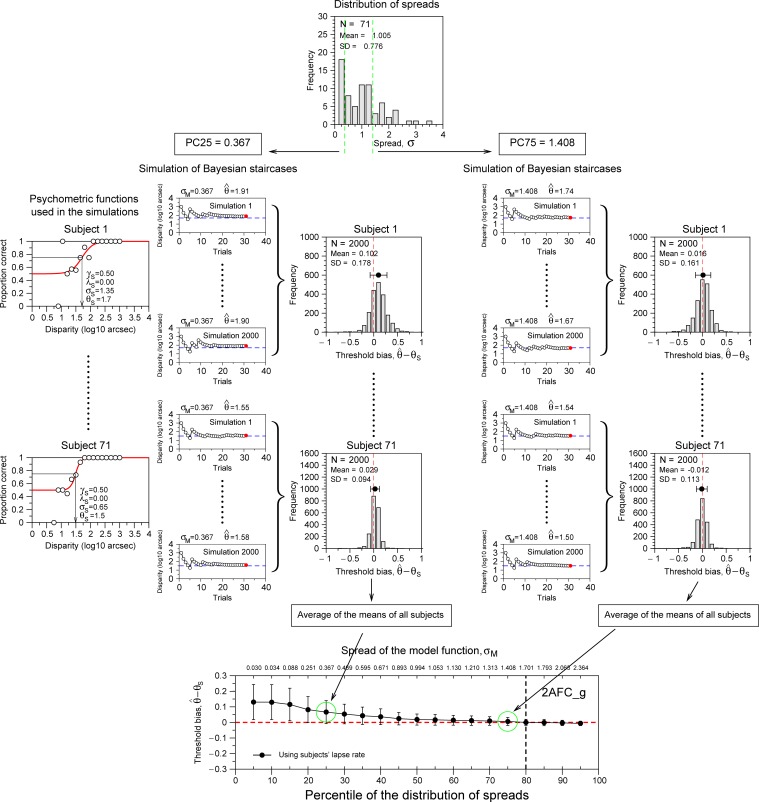
Description of the procedure for obtaining the results presented in Fig 8. This Fig describes procedure to obtain the threshold bias for two percentiles (25 and 75%) of the spreads (σ) obtained in the Experiment 1 (2AFC_g). The top panel shows the histogram of spreads from Fig 4A. The figure shows the example for two percentiles (25 and 75%) of the distribution. To obtain the results of the bottom panel we run 2,698,000 staircases, resulting from 19 percentiles × 71 participants × 2000 staircases of 30 trials. See details in the main text.

In this section we describe the procedure for obtaining the optimal slope or spread (σ_*M*_) value of the model function in order to measure disparity thresholds with small bias and standard deviation when using Bayesian staircases. To do this, we are going to use the spreads, the thresholds, and the lapse rates from the three stereo experiments, two types of task (2AFC and 4AFC), and two types of stereopsis (global and local). The procedure is described in Fig 7. For each experiment we determined 19 different percentiles (from 5 to 95% in steps of 5%) from the distribution of spreads (see example for percentiles 25 and 75% in Fig 7). This gave us 19 different spread values from each experiment.

**Fig 8:**
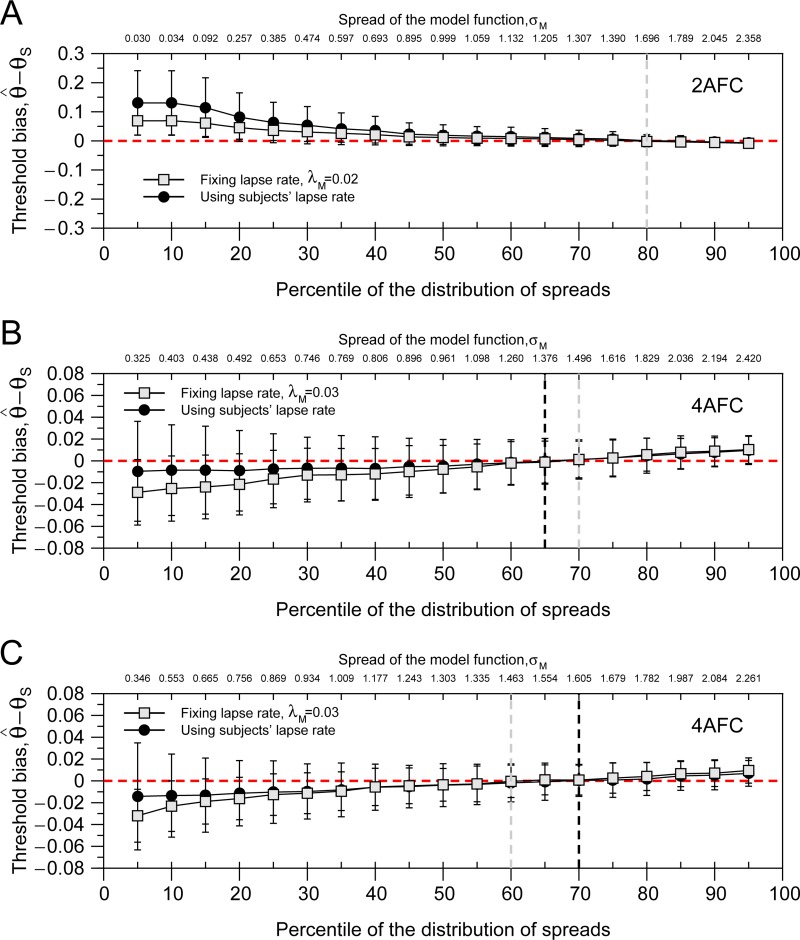
Simulation results using the distribution of spreads from Experiments 1 (A), 2 (B), & 3 (C). Black dots show the threshold bias using for the model function the lapse rate of the subjects. Gray squares show the results for fixed lapse rates of the model function. For 2AFC it was λ_*M*_ = 0.02 (λ* = 0.04) and for 4AFC, λ_*M*_ = 0.03 (λ* = 0.04). The vertical dashed lines mark the spread value (σ_*M*_) or the percentile of the distributions of spreads where the absolute value of the product between SD and Threshold Bias is minimum. Grey dashed line is for condition where model lapse rate (λ_*M*_) was fixed. Note that the range of values of y-axis is different for the three panels.

Then, we used each spread value as the spread of the model function (σ_*M*_,) to run Monte Carlo simulations. For these simulations we used the empirical data (using subject’s thresholds θ_*S*_, spreads σ_*S*_, and lapse rates λ_*S*_) from our experiments in order to model the subject’s psychometric function. In the simulations, the subject responses were replaced by a pseudorandom binary number generator in which the probability of a correct response was read off from the modelled subject’s psychometric function evaluated at the disparity presented on each trial. For each subject we run 2000 simulations using Bayesian staircases of 30 trials (see characteristics of the procedure in S1 Appendix (section A2)). We computed the threshold bias subtracting the estimated threshold and the empirical subject’s threshold ($\hat{\theta }-\theta_{S}$) (Bayesian staircases converged into the probability 0.75 as in the psychophysical experiments) and then, we computed the mean of the 2000 bias estimations. Finally, we average the means bias for all participants (e.g. for Experiment 1, 71 subjects). Thus, one single dot represented in the bottom panel of Fig 7 (or Fig 8A) was obtained by running 142,000 staircases, made up of 1 percentile (i.e. one spread, σ_*M*_) × 71 subjects × 2000 Bayesian staircases.

## Results of the simulations

Fig 8 shows the results of the simulations using the data of the three experiments. We run two conditions: A) black dots, using the lapses from the subjects in the model function; in this condition the only difference between the model function and the subject’s psychometric function was the spread value; B) grey squares, representing the more realistic condition where the individual lapse rates are unknown and so results are obtained for a fixed lapse rate of the model function. For 2AFC we used λ_*M*_ = 0.02 and for 4AFC, λ_*M*_ = 0.03 (both corresponding to λ* = 0.04). We used higher lapse rates than the empirical ones because this produces lower bias and standard errors when using Bayesian staircases [5].

Fig 8A shows the results for Experiment 1 (2AFC_g). In general, threshold estimations are less biased when the model function uses fixed lapse rate (gray squares), but this is only evident for small spread values (i.e. large slope values). The results show a clear pattern: threshold bias and standard deviation are reduced with increasing spread values (i.e. reducing slope values) of the model function. In order to decide what is the optimal spread, we compute the absolute value of the product of the bias and the standard deviation. The vertical dashed lines mark the spread (σ_*M*_) where this product is minimum. In this case, for the 2AFC task, the spread that will produce smaller threshold bias and standard deviation when using a Bayesian staircase for both conditions (fixed lapse rate and subject’s lapse rate) is σ_*M*_ = 1.701 log_10_ arcsec (percentile 80%).

Fig 8B shows the results for Experiment 2 (4AFC_g). In this case, we found an opposite result to the one presented in Fig 8A: this is, threshold estimations are more biased when the model function uses fixed lapse rate (gray squares), but again, this is only evident for small spread values (i.e. large slope values). For fixed lapse rate the optimal spread was σ_*M*_ = 1.496 log_10_ arcsec (percentile 70%); and when we use the subject’s lapse rate, it is σ_*M*_ = 1.376 log_10_ arcsec (percentile 65%). Note that in practical terms this condition is very difficult to happen in empirical studies, we usually don’t know the value of the subject’s lapse rate. However, this simulation tells us that the percentiles for optimal spread are almost independent of the subject’s lapse rate.

Fig 8C shows the results for Experiment 3 (4AFC_l). In this case, as in Experiment 2 (4AFC_g), threshold estimations are more biased when the model function uses fixed lapse rate (gray squares). For fixed lapse rate the optimal spread was σ_*M*_ = 1.463 log_10_ arcsec (percentile 60%); and for the subject’s lapse rate it is σ_*M*_ = 1.605 log_10_ arcsec (percentile 70%). We also found very similar results (results not shown) using Bayesian staircases of 100 trials and also using a probability of correct responses of 0.7.

In summary, the simulations using the empirical data of the experiments show that threshold bias and standard deviation are reduced when spread values correspond to percentiles of the distribution of spreads between 60 and 80%. These percentiles do not depend on the lapse rate of the subjects. Selecting spreads below the percentile 60% introduce bias in all Experiments and spreads higher than 80% introduce a small bias in the Experiments using 4AFC tasks (see left part of Fig 8B and 8C).

## Comparing our procedure with different Bayesian adaptive procedures

In addition to the Bayesian adaptive procedures which simultaneously estimate threshold and the slope of the psychometric function, for example, ZEST 2D [12], MUEST [11], or Psi [13], recently adaptive procedures have been developed that allow us to estimate even more parameters (threshold, slope, and lapse rate) of the psychometric function, such as Psi-marginal [14] or QUEST+ [15]. However, to estimate two or more parameters in an efficient way (small bias and standard deviation) requires more trials than to estimate one single parameter. In clinical applications this can be problematic. For example, our laboratory is designing a stereo test to be run on a 3D tablet [31, 32] in order to measure the stereoacuity in small children, so it is fundamental to obtain efficient stereo thresholds with a small number of trials.

In this section, our objective is to compare our proposed procedure that we will call “ZEST optimal *σ*” with five Bayesian adaptive procedures using Monte Carlo simulations. We tested different versions of the ZEST 2D, Psi, and Psi-marginal procedures. We tested two versions of the ZEST 2D; in one version we estimated two parameters (threshold and slope; “ZEST 2D *θ*,*σ*”); in the other version we only estimate the threshold (“ZEST 2D *θ*”). We tested the original Psi method to estimate threshold and slope (Psi *θ*,*σ*) and we also tested three configurations of the Psi-marginal: one configuration where we estimated the threshold and the slope while marginalizing the lapse rate (Psi *θ*,*σ*(*λ*)), a second configuration where we estimated the threshold only which marginalizing the slope and the lapse rate (Psi *θ*,(*σ*,*λ*)), and a third configuration where we estimated the threshold, marginalize the slope, and fixed the lapse rate (Psi *θ*(*σ*)). The details of these seven procedures are described in S1 Appendix (section A3).

**Fig 9:**
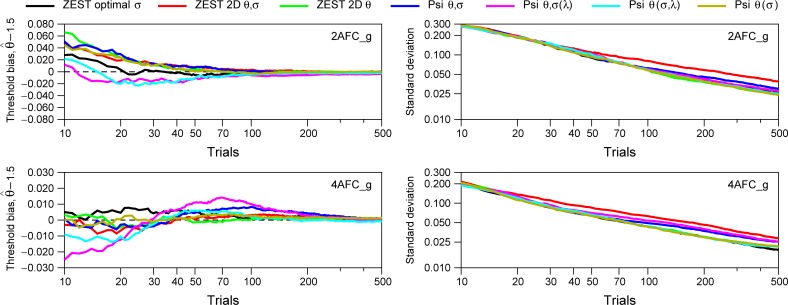
Simulation results for seven Bayesian procedures (Gaussian prior for spreads). Each line corresponds to 1000 simulations for different number of trials for one simulated subject. The parameters of the modelled subject were *σ*_*S*_ = 1 (i.e.*β* = 7.327); *α*_*S*_ = 1.488 (2AFC) or *α*_*S*_ = 1.387 (4AFC); *π* = 0.75; *γ*_*S*_ = 0.5 (2AFC) or *γ*_*S*_ = 0.25 (4AFC); *λ*_*S*_ = 0.02 (2AFC) or *λ*_*S*_ = 0.03 (4AFC); *δ*_*S*_ = 0.012 (2AFC) or *δ*_*S*_ = 0.018 (4AFC), and threshold of *θ*_*S*_ = 1.5 log_10_ arcsec. Left panels show the mean of the threshold bias ($\hat{\theta }-1.5$), and right panels show the standard deviation of the distribution of the threshold bias. For all procedures, for the prior probability distribution of spreads, we have assumed a Gaussian distribution with mean and standard deviation obtained from the data shown in Fig 4A (2AFC_g; mean = 1.005, SD = 0.776) and Fig 4B (4AFC_g; mean = 1.186, SD = 0.740). Note that the y-values for Threshold bias are different for 2AFC and 4AFC tasks.

**Fig 10:**
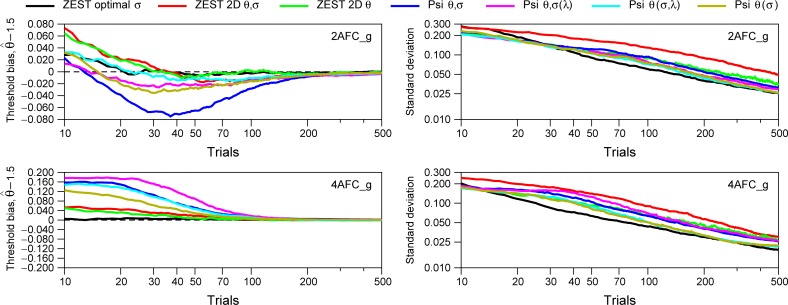
Simulation results for seven Bayesian procedures (uniform prior for spreads). Each line corresponds to 1000 simulations for different number of trials for one simulated subject. The parameters of the modelled subject were *σ*_*S*_ = 1 (i.e.*β* = 7.327); *α*_*S*_ = 1.488 (2AFC) or *α*_*S*_ = 1.387 (4AFC); *π* = 0.75; *γ*_*S*_ = 0.5 (2AFC) or *γ*_*S*_ = 0.25 (4AFC); *λ*_*S*_ = 0.02 (2AFC) or *λ*_*S*_ = 0.03 (4AFC); *δ*_*S*_ = 0.012 (2AFC) or *δ*_*S*_ = 0.018 (4AFC), and threshold of *θ*_*S*_ = 1.5 log_10_ arcsec. Left panels show the mean of the threshold bias ($\hat{\theta }-1.5$), and right panels show the standard deviation of the distribution of the threshold bias. For all procedures, for the prior probability distribution of slopes, we have assumed a uniform distribution. Note that the y-values for Threshold bias are different for 2AFC and 4AFC tasks.

**Fig 11:**
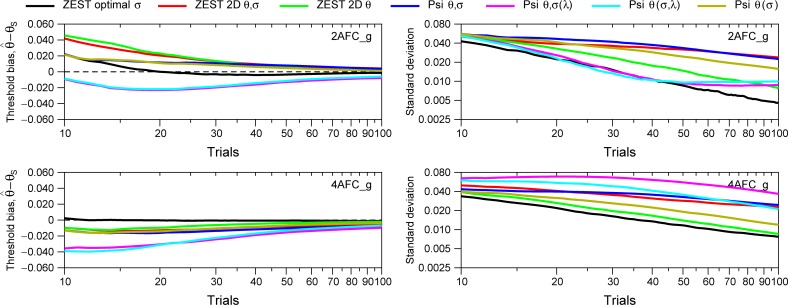
Simulation results for seven Bayesian procedures using the empirical data for the modelled subjects (Gaussian prior for spreads). Left panels show the average of the threshold bias of all subjects. For each subject (out of 71 for 2AFC_g and 68 for 4AFC_g) we run 1000 simulations for different number of trials. Right panels show the standard deviation of the distribution of the threshold bias. For all procedures, we have assumed a Gaussian distribution for the prior probability distribution of spreads, with mean and standard deviation obtained from the data shown in Fig 4A (2AFC_g; mean = 1.005, SD = 0.776) and Fig 4B (4AFC_g; mean = 1.186, SD = 0.740).

**Fig 12:**
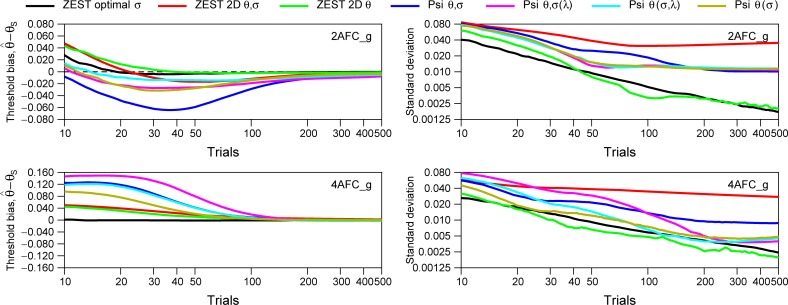
Simulation results for seven Bayesian procedures using the empirical data for the model (uniform prior for spreads). Left panels show the average of the threshold bias of all subjects (i.e. estimated threshold minus the subject’s threshold). For each subject (out of 71 for 2AFC_g and 68 for 4AFC_g) we run 1000 simulations up to 500 trials. Right panels show the standard deviation of the distribution of the threshold bias. We have assumed a uniform distribution for the prior probability distribution of slopes for all procedures. Note that the y-values for Threshold bias are different for 2AFC and 4AFC tasks.

In order to compare all Bayesian procedures, we have performed two types of Monte Carlo simulations: a) using a single model assuming a standard observer based in our data (see Figs 9 and 10) and, b) using different models with the parameters of the empirical data (thresholds, slopes, and lapses) obtained from the experimental conditions 2AFC_g and 4AFC_g (see Figs 11 and 12).

We also have performed simulations for 2AFC and 4AFC tasks. For each task we have used two types of prior probability distribution of slopes, the uniform distribution (i.e. non-informative distribution) and a Gaussian distribution based in our data. Although there are hierarchical adaptive approaches like HADO [33] that allow us to construct informative priors based on data of observers that have previously performed the tasks, in order to simplify the comparisons and use the same priors for each method, we have assumed a Gaussian distribution with mean and standard deviation obtained from the data shown in Fig 4A (2AFC_g) and Fig 4B (4AFC_g). For the thresholds, we have assumed a uniform prior distribution for all simulations. For ZEST optimal *σ*, (see details in S1 Appendix (section A2)), the *σ*_*M*_ used in the simulations for the model functions was *σ*_*M*_ = 1.7 log_10_ arcsec (slope of *β*_*M*_ = 4.3) for 2AFC task, and *σ*_*M*_ = 1.5 log_10_ arcsec (*β*_*M*_ = 4.9) for 4AFC. The guess and lapse rates used for the model functions were *γ*_*M*_ = 0.5 and λ_*M*_ = 0.02 for 2AFC and *γ*_*M*_ = 0.25 and λ_*M*_ = 0.03 for 4AFC. The details about the parameters used in the other Bayesian procedures can be seen in S1 Appendix (section A3).

Figs 9 and 10 show the results of the simulations comparing our suggested procedure with the other five procedures for a simulated subject (i.e. a theoretical subject). Fig 9 show the simulation results assuming a Gaussian distribution for prior distribution of spreads based on the results from Fig 4 (for 2AFC, mean = 1.005, SD = 0.776; for 4AFC, mean = 1.186, SD = 0.740). Fig 10 shows the simulation results assuming a prior uniform distribution. The choice of prior only affects models which estimate or marginalize spread, thus, the black curves for ZEST optimal spread are the same in both figures. For thresholds, we have used a uniform prior distribution in all simulations. The parameters of the modelled subject were *σ*_*S*_ = 1 (i.e.*β*_*S*_ = 7.327); *α*_*S*_ = 1.488 (2AFC) or *α*_*S*_ = 1.387 (4AFC); *π* = 0.75;*γ*_*S*_ = 0.5 (2AFC) or *γ*_*S*_ = 0.25 (4AFC); *λ*_*S*_ = 0.02 (2AFC) or *λ*_*S*_ = 0.03 (4AFC); *δ*_*S*_ = 0.012 (2AFC) or *δ*_*S*_ = 0.018 (4AFC), and threshold of *θ*_*S*_ = 1.5 log_10_ arcsec. We have chosen these parameters because, according to our data, they represent rather well a standard subject in a stereoacuity task (see these two psychometric functions in Fig 1A, right panel). Note that our ZEST optimal *σ* makes incorrect assumptions about this model observer: it assumes a spread of 1.5 log_10_ arcsec, not the true value of 1 log_10_ arcsec. The left panels of Figs 9 and 10 show the mean of the threshold bias ($\hat{\theta }-1.5$), and the right panels show the standard deviation of the distribution of threshold bias. Each line corresponds to 1000 simulations of each Bayesian procedure for different number of trials, thus, the mean and the SD for each trial was obtained from 1000 threshold bias.

Results from Figs 9 and 10 show that the threshold bias is smaller for 4AFC than 2AFC for the same number of trials, in agreement with previous results [34]. Fig 9 shows that all procedures increase in precision (i.e. lower threshold bias) with increasing number of trials. For 2AFC task, our suggested procedure (black line, ZEST optimal *σ*) shows the smallest bias and smaller SD in general and for small number of trials (between 20 and 50) in particular. For 4AFC task, for trials greater than 30, all procedures give similar results where Psi *θ*,*σ*(*λ*) (pink line) shows the worst performance in terms of threshold bias, and Psi *θ*(*σ*) (brown line) shows the smallest threshold bias. Note that the black lines corresponding to Zest optimal *σ* are the same for Figs 9 and 10 given that we are not using a prior distribution for *β* for this method. Note also that in empirical testing, the SD of threshold’s estimates obtained with Bayesian staircases longer than 70 trials may not decrease with the increasing number of trials [35].

Fig 10, when a uniform distribution is assumed for the slope, shows that for both 2AFC and 4AFC our procedure (black line) shows the lowest threshold bias and SD for trials greater than 20. Other good options would be the method ZEST 2D *θ* (green line) and Psi *θ*,(*σ*,*λ*) (cyan line). Note that, although the method ZEST 2D *θ*,*σ* (red) shows small threshold bias, the SD is the highest of all procedures.

In Figs 9 and 10, for ZEST optimal *σ*, we chose parameters such that the model function underestimated the slope of the simulated observer and assumed the correct lapse rate. We also assumed the correct lapse rate for ZEST 2D *θ*, ZEST 2D *θ*,*σ*, Psi *θ*,*σ*, and (Psi *θ*(*σ*)). These choices may favor these approaches over the configurations of Psi-marginal Psi *θ*,*σ*(*λ*) and Psi *θ*,(*σ*,*λ*). In real life we will encounter observers with unusually low slopes and with lapse rates different from those we assumed. To assess performance under these conditions, Figs 11 and 12 show the simulation results using different modelled subjects with the parameters of the empirical data (thresholds, slopes, and lapses) obtained from the experimental conditions 2AFC_g and 4AFC_g. Fig 11 shows the simulation results assuming a Gaussian distribution for prior distribution of spreads based on the results from Fig 4, and Fig 12 shows the simulation results assuming a prior uniform distribution. Like in Figs 9 and 10, for disparity thresholds, we have used a uniform prior distribution in all simulations.

The left panels of Figs 11 and 12 show the average of the threshold bias of all subjects. For each subject (out of 71 for 2AFC_g and 68 for 4AFC_g) we have run 1000 simulations for different number of trials (between 10 and 100 and between 10 and 500 for Fig 12). The right panels show the standard deviation. Note that, like in Figs 9 and 10, the black lines corresponding to Zest optimal *σ* are the same for Figs 11 and 12 given that we are not using a prior distribution for *β* for this method.

Results from Fig 11 show that ZEST optimal *σ* (black line) has the lowest threshold bias for both tasks and is constant from trials higher than 20. The rest of the procedures show a higher threshold bias that is very pronounced at small number of trials. The procedure ZEST optimal *σ* also shows the smaller standard deviation for all number of trials tested. Fig 12, when assuming a uniform distribution for the slope, shows similar results although in this case, for the 4AFC task, the procedure ZEST 2D *θ* (green line) also shows very small bias and standard deviation. For small number of trials (< 30) ZEST optimal *σ* is recommended. Thus, in general, if the number of trials is small and we have knowledge about the distribution of slopes, we recommend the procedure “ZEST optimal *σ*”, however, taking into account that the distribution of the slopes in an experiment is probably unknown, the procedure ZEST 2D *θ* is also a good candidate if the experimenter is only interested in estimating thresholds and assumes the uniform prior distribution for the spreads, and the procedure Psi *θ*(*σ*) is also a good candidate for 4AFC tasks if the experimenter is only interested in the threshold.

**Fig 13:**
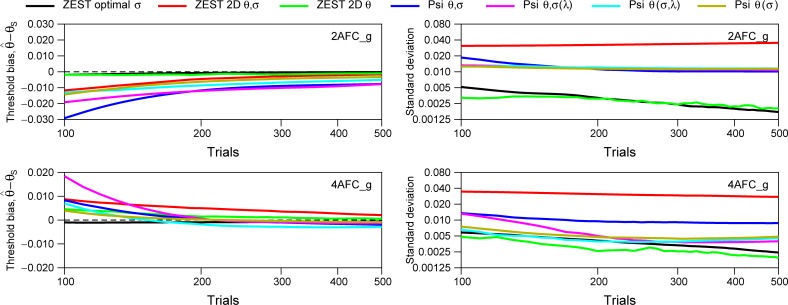
Results presented in Fig 12 but from 100 to 500 trials in order to show the differences between these methods for high number of trials.

Fig 13 shows the simulations results presented in Fig 12 but from trials 100 to 500 to easily compare the methods for a high number of trials. Results show that threshold bias is negligible from 200 trials being stronger for Psi (blue line) and Psi-marginal (cyan and pink line) in 2AFC and ZEST 2D *θ*,*σ* (red line) for 4AFC. In terms of standard deviation, for 2AFC, our procedure ZEST optimal *σ* and ZEST 2D *θ* show the smallest standard deviation followed by Psi-methods and ZEST 2D *θ*,*σ*. For 4AFC, again, ZEST optimal *σ* and ZEST 2D *θ* show the smallest standard deviation. Psi *θ*,(*σ*,*λ*) (cyan line) shows very similar standard deviation between 100 and 300 trials. In general, for 4AFC, both versions of Psi-marginal shown here, work better than the classic Psi method. It is important to note that ZEST optimal *σ* still shows smaller bias and standard deviation than the Psi-marginal procedures tested here because we are using the optimal spread for the population, however, these Psi-marginal procedures are very good when we have no idea about the distribution of slopes, and we can run more than 100 trials in the experiment. Finally, ZEST 2D *θ*,*σ*, showed smaller bias than Psi procedures although higher standard deviation.

## Discussion

Parametric procedures like Bayesian staircases require a priori decisions about different parameters of the assumed psychometric function or model function (e.g. shape, slope, convergence probability and lapse rate). Simulation studies have shown that mismatches between the subject psychometric function and the model function produce threshold bias [3–5]. In this study, our aim was to find the optimal spread value (inversely related to the slope) of the model function in order to estimate disparity thresholds with the smallest bias and standard deviation. In particular, we wanted to find this value for measuring disparity thresholds for global stereopsis (or cyclopean) and local stereopsis (feature or contour-based). We also wanted to know if this value changes with the psychophysical task (i.e. 2AFC vs. 4AFC). To achieve this, we needed to know the distribution of real slopes (i.e. spreads) for the general population for these tasks and types of stereopsis. Therefore, we ran three experiments, 2AFC_g with global stereopsis, 4AFC_g with global stereopsis, and 4AFC_l with local stereopsis where we tested in total 260 participants. For each participant we ran an adaptive weighted one-up one-down staircase procedure with 80–120 trials and we fitted a logistic psychometric function (described in the S1 Appendix (section A1)) to the probabilities of correct detection in order to estimate stereoacuity thresholds, spread, and lapse rates. Results from these experiments are shown in Figs 4, 5 & 6. We found that disparity thresholds were around 1.5 arcsec for all experiments. With regard to the spread values (inversely related with the slope), we found differences between 2AFC_g and 4AFC_l, with spread values smaller for 2AFC_g. No significant differences were found between the two 4AFC experiments. We also found that for the three experiments, the spread values did not correlate with age. Finally, an analysis of the lapse rates showed a similar probability of making a lapse in all experiments (between 0.02 and 0.03, a percentage of 2–3%).

We have developed a new method in order to estimate the optimal slope or spread (see Fig 7), where we simulate millions of Bayesian staircases using the empirical spreads, thresholds and lapse rates of our participants as model subject’s psychometric functions. The spreads used in the assumed psychometric function or model function corresponded to percentiles (between 5 and 95% in steps of 5%) of the empirical distributions of spreads. Fig 8 shows the results of the simulations. These results show a clear pattern: bias and errors (i.e. standard deviation) are reduced when the spread value is located between the percentile 60–80% of the distributions of spreads. These percentiles work for three different distributions of spreads. We simulated two conditions, one where the model lapse rate was the same as the subject, and the other (the more realistic one) where the lapse rate was fixed to a particular value. We found that the two conditions differ only when the spread value is very small; however, they are very similar when the spreads are higher than the 60% percentile of the distribution of spreads. Thus, for the first time, we have been able to estimate the optimal spread values for different types of stereopsis (e.g global and local) and tasks (e.g. 2AFC and 4AFC). These values can be used with Bayesian staircases to estimate thresholds with the smallest bias and standard deviation independently of the age of the participant.

It is important to note that there are Bayesian methods that can be used to estimate the threshold and the slope simultaneously, or just the threshold but leaving the slope as a nuisance parameter [11–15]. Here, running Monte Carlo simulations with the empirical data as modelled subjects, we have compared our suggested procedure (ZEST using the optimal slope, “ZEST optimal *σ*”) with six Bayesian methods, two based on the ZEST 2D [12], the Psi method [13], and three configurations of Psi-marginal [14]. Our simulation results (see Figs 11 and 12) show that, in general, ZEST optimal *σ* has lower threshold bias and standard deviation for 2AFC and 4AFC tasks than the rest of the Bayesian procedures tested. This difference in threshold bias is very pronounced at small number of trials. For example, Figs 10 (4AFC) and 12 (4AFC) shows that Psi methods have a strong bias for small number of trials (between 20 and 30), meaning that they systematically tend to overestimate thresholds by a factor of 1.38–1.45 (e.g. a threshold of 100 arcsec will be incorrectly estimated as 145 arcsec). In addition, the standard deviation for 20–30 trials is 0.04–0.02, corresponding to multiplying or dividing by a factor of 1.05–1.1. For Zest optimal *σ* in the range 20–30 trials, the bias factor is 0.998 and the standard deviation range is 0.017–0.013, that corresponds to a factor of 1.03–1.04. We have to acknowledge that the simulations were performed using the same distributions that were used to estimate the optimal slope, so it is expected to behave with the smallest threshold bias, however, it is also expected that is the distribution of spreads is approximately known, the use of ZEST with the optimal *σ* will produce unbiased thresholds.

Thus, if the number of trials used for estimating the threshold is small (< 30), we recommend using ZEST optimal *σ*. This procedure is currently being used in a 3D software dedicated to estimate the stereoacuity (ASTEROID, [31, 32]). However, if the optimal slope is unknown, and the experimenter assumes a uniform distribution for the slope, our results shows that the procedure ZEST 2D *θ* is a very good candidate followed by Psi-marginal with the configuration Psi *θ*(*σ*) or Psi *θ*,(*σ*,*λ*).

Our recommendation for choosing the optimal spread is to overestimate the subject’s spread (i.e. underestimate the subject’s slope). If the distribution of spreads is known, choose a spread value between the percentile 60 and 80% approximately. We have shown that these percentiles work for three different experiments with different distributions of spreads, thresholds, and lapse rates, thus, our results could be extended to other type of thresholds if the distributions of spreads are known. However, we recommend using the procedure described in our Fig 7 to get more precision. For our particular stereoacuity experiments, we recommend using ZEST (as described in S1 Appendix (section A2)), assume a cumulative logistic distribution for the model function, use fixed lapse rates, and the spread value of the model function around the value *σ*_*M*_ = 1.7 log_10_ arcsec (corresponding to a factor of 50 in arcsec, or a slope of *β*_*M*_ = 4.3 /log_10_ arcsec) for 2AFC experiments, and around *σ*_*M*_ = 1.5 log_10_ arcsec (a factor of 32 in arcsec, *β*_*M*_ = 4.9 /log_10_ arcsec) for 4AFC.

### Limitations of the study

These recommendations are of course not perfect; for example, they neglect the possible effects of changes in an observer’s psychometric function during the course of the experiment, or spatial biases towards a particular location. Further work would be needed to assess these. Another limitation of our study is that for the simulations we are only using three distributions of spreads (also three distributions of thresholds and lapse rates), obtained with a psychophysical method that is far from perfect (i.e. more trials would be needed to obtain a better estimation of the parameters, increasing the range of age of the participants would be recommended). Therefore, these distributions do not reflect the exact distributions of spreads, thresholds, and lapse rates. However, given that simulations for the three experiments shows similar results our recommendations would be useful in this field in order to estimate stereo thresholds. Future directions should include simulations assuming different shapes for the distribution of spreads in combination with different distributions of thresholds and lapse rates, will give a more precise answer and more generalizable recommendations to other visual modalities.

## Supporting information

S1 Appendix Description of the psychometric function used in this research and the Bayesian procedures used in the simulations. (PDF)

S1 Dataset The data presented in this paper. (XLSX)
