# Supplementary material for: Efficient estimation of stereo thresholds: What slope should be assumed for the psychometric function?
Source: PLoS One. 2020 Jan 2;15(1):e0226822. doi: 10.1371/journal.pone.0226822 (PMC6939937; doi:10.1371/journal.pone.0226822)
Supplement: S1 Appendix — (PDF) [file pone.0226822.s001.pdf]

Supplementary Information

Efficient estimation of stereo thresholds: what slope should be assumed for the psychometric function?

Ignacio Serrano-Pedraza, Kathleen Vancleef, William Herbert, Nicola Goodship, Maeve Woodhouse & Jenny C. A. Read

Appendix

In this appendix we describe the psychometric function used in this research and the Bayesian procedures used in the simulations.

A1. Logistic function

In all fittings and simulations presented in this research we have used the cumulative Logistic distribution (see Fig. A1) (Pentland, 1980; Emerson, 1986; Madigan & Williams, 1987; Serrano-Pedraza et al., 2016b) adapted from García-Pérez, (1998, see his appendix A):

$$\psi(x;\alpha,\beta,\lambda,\gamma)=\gamma+\frac{1-\lambda-\gamma}{1+\exp[\beta(\alpha-x)]} \tag{A1}$$

where x is the disparity (arcsec) in decimal logarithmic units ($\log_{10}$(arcsec)); γ, is the guess rate (i.e γ_{2AFC}=0.5 and γ_{4AFC}=0.25); λ is the probability of responding incorrectly as a result of a lapse (Kingdom & Prins, 2010), $\lambda=\lambda^*(1-\gamma)$, where λ^* is the probability of making a lapse. Thus, for the same probability of making a lapse, the value of λ will change depending if the task is 2AFC or 4AFC. The parameter β controls the steepness of the psychometric function. This is related to the spread (σ) as follows:

$$\beta=\frac{2}{\sigma}\ln\left[\frac{1-\lambda-\gamma-\delta}{\delta}\right]. \tag{A2}$$

We define the spread (σ) or region of support of the psychometric function as

$$\sigma=\psi^{-1}(1-\lambda-\delta)-\psi^{-1}(\gamma+\delta) \tag{A3}$$

where δ, is used to calculate σ with a desired central range ("CR", in percentage) of the psychometric function:

$$\delta=\frac{100-\mathrm{CR}}{200}\times(1-\lambda-\gamma). \tag{A4}$$

Thus, the spread of the psychometric function is the range of stimulus intensities (in log-decimal units) (the range being set by lapse rate and guessing rate). Substituting (A4) into (A2), we obtain

$$\beta = \frac{2}{\sigma} \ln \left(\frac{100 + \mathrm{CR}}{100 - \mathrm{CR}} \right).$$

(A5)

Thus, the relationship between the slope parameter β and the σ parameter is independent of the chance performance rate or of the observer's lapse rate. We used CR=95 in all simulations and fittings so the spread value corresponds to the 95% range of stimulus intensities for a particular lapse rate and guessing rate (see Fig. A1, left panel), in which case:

$$\beta = 7.327/\sigma. \quad \text{(A6)}$$

The location parameter α is defined as follows:

$$\alpha = \theta + \frac{1}{\beta} \ln \left[\frac{1 - \lambda - \pi}{\pi - \gamma} \right],$$

(A7)

where π, is the probability of correct responses associated with the threshold θ (in $\log_{10}$ units). We used π=0.75 for both, 2AFC and 4AFC tasks and for the fittings and the simulations.

Examples of the Logistic function with different parameter values can be seen in Fig. A1.

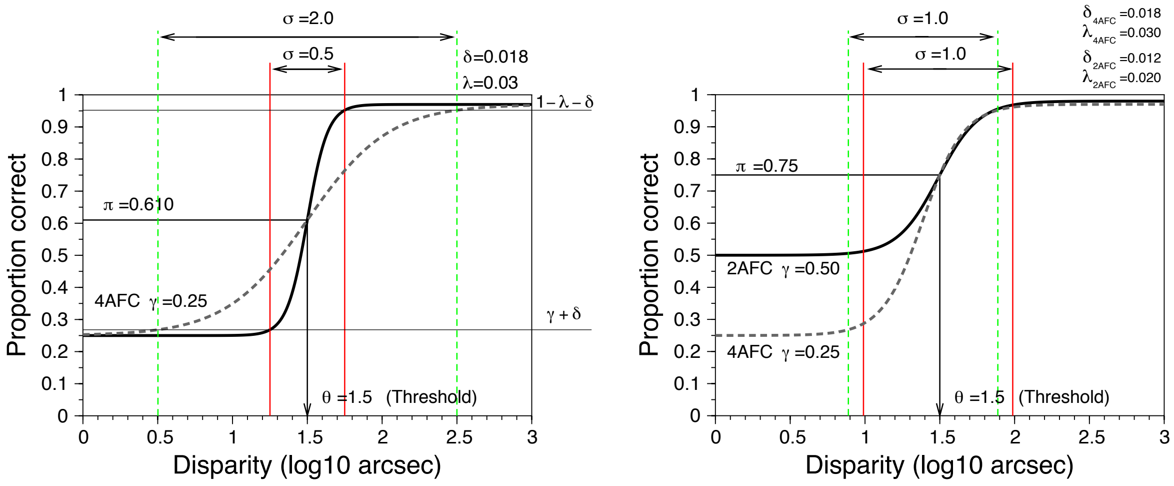

Fig. A1. Examples of the Logistic psychometric function with different parameter values. Width of the central range (CR) CR= 95%, σ = (7.327/ β). In all examples, the threshold value is $\theta = 1.5$ $\log_{10}$ arcsec. Left panel shows two examples with different spread values. Black line ($\beta = 14.654$ ($\sigma = 0.5$); $\alpha = 1.5$; $\pi = 0.61$; $\gamma = 0.25$; $\lambda = 0.03$; $\delta = 0.018$); grey dashed line ($\beta = 3.66$ ($\sigma = 2$); $\alpha = 1.5$; $\pi = 0.61$; $\gamma = 0.25$; $\lambda = 0.03$; $\delta = 0.018$). Right panel shows examples for 2AFC and 4AFC with the same spreads and thresholds. Black line ($\beta = 7.327$ ($\sigma = 1$); $\alpha = 1.488$; $\pi = 0.75$; $\gamma = 0.5$; $\lambda = 0.02$; $\delta = 0.012$); grey dashed line ($\beta = 7.327$ ($\sigma = 1$); $\alpha = 1.387$; $\pi = 0.75; \gamma = 0.25$; $\lambda = 0.03$; $\delta = 0.018$).

A2. Description of the ZEST optimal σ staircase procedure

In this section we describe the characteristics of the Bayesian staircases used in the simulations. The procedure is similar to ZEST (King-Smith, et al., 1994). If the spread value used in this method is the optimal one, then we will call this procedure ZEST optimal σ:

1) *Prior distribution.* We used a non-informative prior for the thresholds or a prior of ignorance (Treutwein, 1995). In particular, we used the uniform distribution $u(x)$ (Emerson, 1986). Where, $u(x) = 1$ for $x \in [0, 3.556]$ in steps of 0.0036, where x corresponds to the disparity values in $\log_{10}$ (arcsec). For the simulations comparing procedures we used the range $x \in [0.477, 3.176]$ in steps of 0.05508 (the same range that was used for ZEST 2D, Psi and Psi-marginal in the comparison study).
2) *Model function.* This is the assumed subject's psychometric function. We used the Logistic function (Pentland, 1980; Emerson, 1986; Madigan y Williams, 1987) described in section A1 of this Appendix (see equation A1). Odd symmetric functions about threshold are recommended in simulation studies using Bayesian procedures (Alcalá-Quintana y García-Pérez, 2004). Here we will call the model function $M(x, \theta)$ where θ is the disparity value that is presented in each trial that will be the value of the disparity threshold in the last trial. The model function has the slope parameter β_M defined as $\beta_M = -7.327/\sigma_M$; the spread value σ_M; the position parameter α_M; the guess rate γ_M, and the lapse rate λ_M.
3) *Staircase updating.* On the first trial we forced the staircase to start at a high level of the stimulus, $\theta_1 = 3$ (1000 arcsec). After the first trial, the posterior probability distribution is the uniform $P_1(x) = u(x)$, then, from the second trial the posterior distribution is obtained and updated this way:
$P_{i+1}(x) = P_i(x)M(x, \theta_i)^C[1 - M(x, \theta_i)]^I,\ i \in [1, n]$,
where C=1 and I=0 after a correct response and I = 1and C = 0 after an incorrect response and *n* is the number of trials.
4) *Placement rule.* The stimulus intensity (i.e. disparity) for each trial was selected computing the mean of the posterior distribution $\theta_i = \overline{P_i(x)}$. Simulation studies have shown that with the same number of trials, the mean minimizes the variance of the threshold estimate and is less biased than using the mode or the median of the posterior probability distribution (Emerson, 1986, King-Smith et al. 1994; Alcalá-Quintana y García-Pérez, 2004). The threshold was obtained from the mean of the final probability distribution $P_{n+1}(x)$.
5) *Stopping rule.* The staircases stopped after a fixed number of trials (Emerson, 1986; Madigan y Williams, 1987; Anderson, 2003).

A3. Bayesian adaptive procedures used in the comparison study

A.3.1 ZEST 2D

This Bayesian adaptive procedure proposed by King-Smith and Rose (1997) is an extension of the original ZEST procedure (King-Smith et al., 1994) and allows to estimate the threshold and the slope of the subject's psychometric function with one staircase. This procedure uses a two-dimensional array of thresholds and slopes (see examples in Fig. A2) that is updated every trial according to Bayes' theorem (see also Watson & Pelli, 1983 and the Appendix of King-Smith et al., 1994). For all simulations we used an array of 50 thresholds × 50 slopes in log values. The range of thresholds (θ) used in the simulations was set from $\log_{10}$(3 arcsec) to $\log_{10}$(1500 arcsec) in steps of 0.05508 log units (this gives a total of 50 thresholds). The range for the slopes (β) was set from $\log_{10}(0.3)$ to $\log_{10}(70)$ in steps of 0.483 log units (this gives a total of 50 slopes). The *staircase updating*, and the *stopping rule* were the same as described in section A2. In particular, for the staircase updating we will use the 2D model functions shown in Fig. A2B for correct responses and Fig. A2C for incorrect responses. The procedure ZEST 2D allows us to choose two options for the *placement rule* (i.e. the selection of the stimulus intensity for the next trial). In the first one, that we will call ZEST 2D θ, the stimulus intensity for each trial is chosen from the mean of the posterior distribution (see equation 6 of King-Smith & Rose, 1997). The slope can also be estimated from the mean of the posterior (see equation 7 of King-Smith & Rose, 1997). A simulation example of this procedure can be seen in Fig. A3. This option is recommended if we are only interested in estimating the threshold because the estimation of the slope is very imprecise.

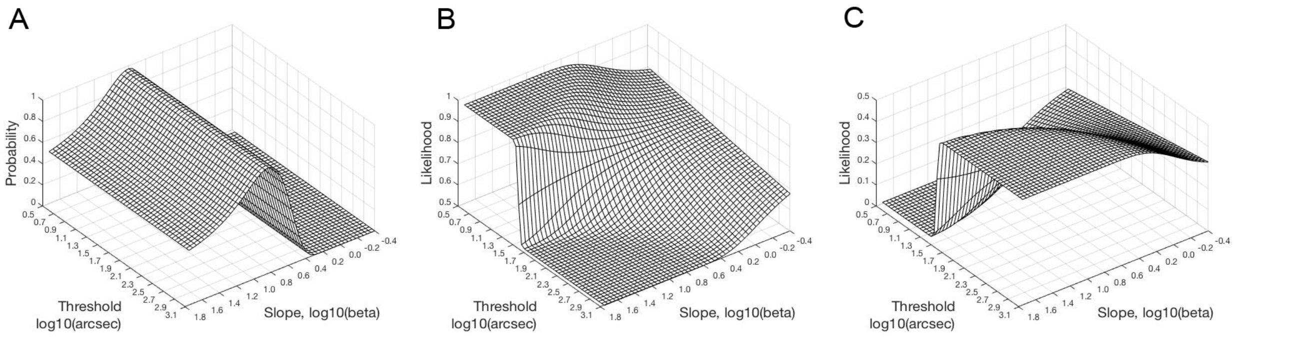

Fig. A2. 3D plots showing an example of one prior distribution and the model functions used in ZEST 2D, Psi, and Psi-marginal. **A.** Prior probability distribution of slopes ($\log_{10}(\beta)$). For the simulations we have used two types of distributions for the slope, the uniform (i.e. non-informative distribution) and a distribution based in our data, in particular, we have assumed a Gaussian distribution with mean and standard deviation obtained from the data shown in Fig. 4A (2AFC_g, this example) and Fig. 4B (4AFC_g). For the thresholds, we have assumed a uniform prior distribution in all simulations. **B.** Two-dimensional model function for correct responses for a 2AFC task ($\gamma_M = 0.5$, $\lambda_M = 0.02$). **C.** Two-dimensional model function for incorrect responses. Both examples are located in the threshold value of θ =1.5 $\log_{10}$ arcsec for $\pi = 0.75$.

The second option for the placement rule (we will call this procedure ZEST *2D* θ, σ) is to present the stimulus intensity at the sweat factors or sweet points (Taylor, 1971; Green, 1990; Green, 1993) for the slope (β). For the Logistic psychometric function there are two β-sweet points (King-Smith & Rose, 1997; Snoeren & Puts 1997; Shen & Richards, 2012).

In order to obtain the probabilities of the psychometric function associated with the maximum slope efficiency, we minimized the expected variance using the equation A2 from Shen & Richards (2012). These probabilities depend on the guess rate (γ) and the lapse rate (λ). Assuming the Logistic function (see our equation A1), for the 2AFC task ($\gamma_M = 0.5$, $\lambda_M = 0.02$) the probabilities were 0.587 and 0.926, and for the 4AFC task ($\gamma_M = 0.25$, $\lambda_M = 0.03$) the probabilities were 0.368 and 0.887.

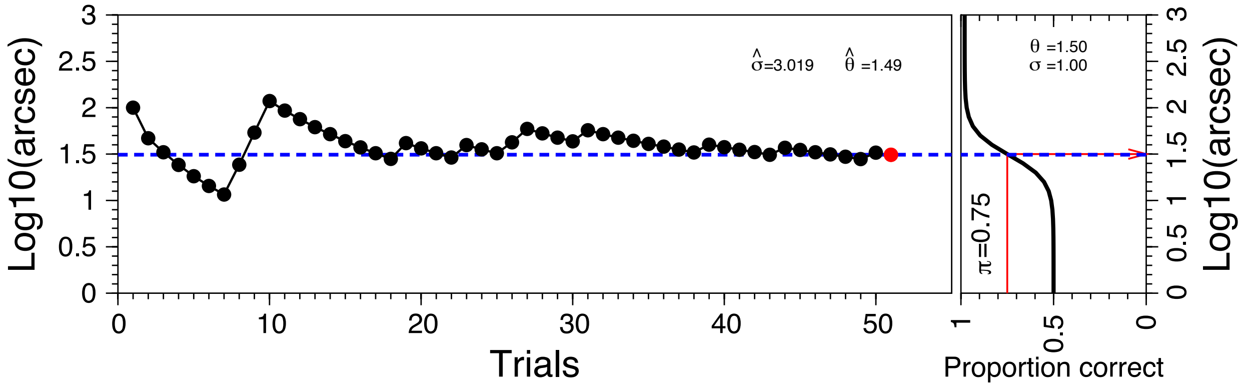

Fig. A3. Simulation example of procedure **ZEST 2D $\boldsymbol{\theta}$**. The left panel shows the stimulus intensities (i.e. mean of the 2D posterior distribution) for each trial (black dots). The red dot corresponds to the final disparity threshold estimate that corresponds to the dashed blue line. The right panel shows the psychometric function (black line) of one modelled subject used in the simulations with a threshold of θ_S =1.5 $\log_{10}$ arcsec for $\pi = 0.75$. The parameters of the modelled subject were $\sigma_S = 1$ (i.e. $\beta_S = 7.327$); $\alpha_S = 1.488$; $\pi = 0.75$; $\gamma_S = 0.5$; $\lambda_S = 0.02$; $\delta_S = 0.012$.

In each trial, the threshold and the slope were estimated from the means of the two-dimensional posterior distribution (see equations 6 and 7 of King-Smith & Rose, 1997), then, after a correct or incorrect response, the 2D model function (see Figs. A2B and A2D) was located in the threshold value that corresponds to $\pi = 0.75$, and the stimulus intensity for the next trial was chosen randomly from one of the two β-sweet points of the Logistic function using the estimated slope. A simulation example of this procedure can be seen in Fig. A4. The upper-leftward panel shows the stimulus intensities (black dots) and the threshold estimates in each trial (red dots). The lower-leftward panel shows the slope estimates (transformed to σ values) (see more details in the legend of Fig. A4).

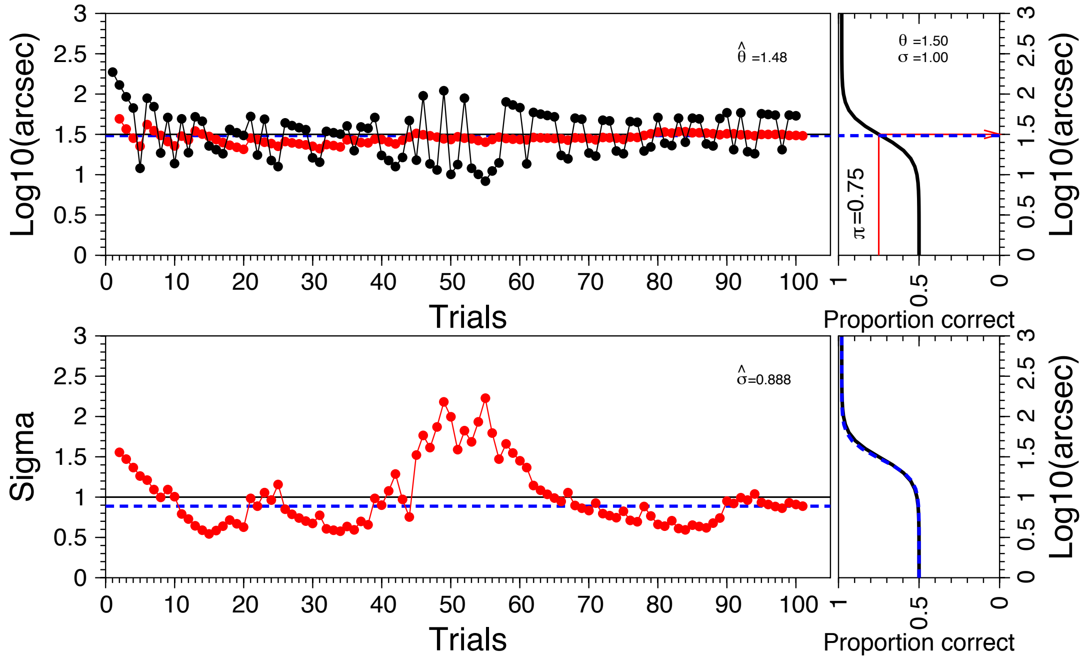

Fig. A4. Simulation example of procedure **ZEST 2D $\boldsymbol{\theta, \sigma}$**. The upper-leftward panel shows the stimulus intensity (i.e. disparity) presented in each trial (black dots). Red dots show the estimated threshold from the mean of the posterior distribution. The last red dot corresponds to the final threshold estimate that corresponds to the dashed blue line. The upper-rightward panel shows the psychometric function (black line) of one modelled subject used in the simulations with a threshold of θ_S =1.5 $\log_{10}$ arcsec for $\pi = 0.75$. The parameters of the modelled subject were $\sigma_S = 1$ (i.e. $\beta_S = 7.327$); $\alpha_S = 1.488$; $\pi = 0.75$; $\gamma_S = 0.5$; $\lambda_S = 0.02$; $\delta_S = 0.012$. The lower-leftward panel shows the estimated slopes (transformed to σ values) from the mean of the posterior distribution. The black line represents the value of the spread of the model ($\sigma = 1$). The last red dot is the final slope estimate that corresponds to the dashed blue line. On the lower-rightward panel, the model psychometric function is represented in black and the psychometric function estimated from the staircase is represented with a dashed blue line with $\gamma = 0.5$ and $\lambda = 0.02$.

A3.2 Psi

This Bayesian adaptive method was proposed by Kontsevich & Tyler (1999) to estimate the threshold and the slope of the psychometric function. Here, we will call this method Psi θ, σ. This procedure uses the same two-dimensional array of thresholds and slopes used by ZEST 2D (see examples in Fig. A2) and the posterior probability distribution is updated according to Bayes' theorem. The range of the thresholds and slopes used here for constructing the two-dimensional array of 50 thresholds × 50 slopes is the same as described for the procedure ZEST 2D (see section A3.1). The *staircase updating*, and the *stopping rule* were the same as described in section A2. However, the *placement rule* in Psi (i.e. the selection of the stimulus intensity for the next trial) is different from ZEST 2D. For Psi, the stimulus intensity that will be presented in the next trial is the intensity that has the minimum expected entropy (using the Shannon entropy) in the posterior distribution that will result for a correct or an incorrect response (Pelli, 1987; see steps 3, 4 & 5 of the Method's section of Kontsevich & Tyler, 1999; or equations 5, 6 and 7 of Watson, 2017). Although Pelli (1987) suggested to calculate the entropy for all future possible trials, Psi method minimizes the expected entropy only at the end

of the next trial. This decision is based on the study of King-Smith et al., (1994) that suggested that minimizing the expected variance of the posterior distributions (an alternative way of selecting the intensity of the next trial) at the end of the next trial (i.e. looking only one trial ahead) was as good as looking two trials ahead. The range of stimulus intensities used to calculate the expected entropy was set from $\log_{10}$(3.16 arcsec) to $\log_{10}$(1000 arcsec) in steps of 0.0862 log units (in total, 30 different stimulus disparities). The estimation of the threshold and the slope is based on computing the mean of the two-dimensional posterior distribution accordingly (see equations 6 and 7 of King-Smith & Rose, 1997). An alternative procedure called QUEST + (Watson, 2017) is similar to Psi but uses the mode of the posterior distribution to estimate the parameters of the psychometric function.

If the slope of the psychometric function is known by the experimenter, Psi method can be used with the version "slope-constrained" Psi (i.e. only one slope parameter is used, so there is no more two-dimensional array). Kontsevich & Tyler (1999) showed that the slope-constrained Psi and ZEST give similar results, thus, our suggested ZEST-optimal σ method will give similar results to the method slope-constrained Psi with the optimal σ.

A simulation example of the method Psi θ, σ is shown in Fig. A5. The upper-leftward panel shows the stimulus intensities and the threshold estimation. The lower-leftward panel shows the spread estimation as a function of the number of trials.

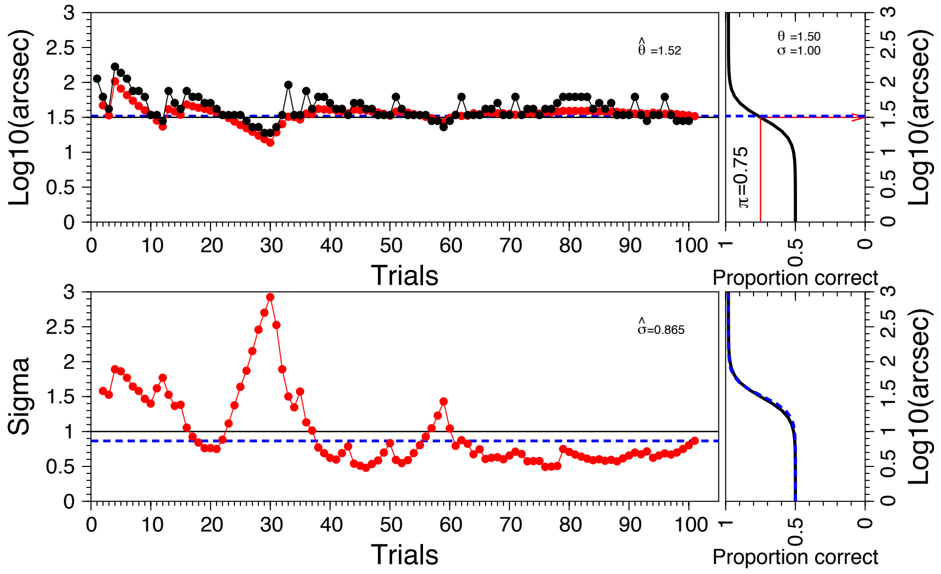

Fig. A5. Simulation example of procedure **Psi $\boldsymbol{\theta, \sigma}$**. The upper-leftward panel shows the stimulus intensity (i.e. disparity) presented in each trial (black dots). Red dots show the estimated threshold from the mean of the posterior distribution. The last red dot corresponds to the final threshold estimate that corresponds to the dashed blue line. The upper-rightward panel shows the psychometric function (black line) of one modelled subject used in the simulations with a threshold of θ_S =1.5 $\log_{10}$ arcsec for $\pi = 0.75$. The parameters of the modelled subject, in this example, were $\sigma_S = 1$ (i.e. $\beta_S = 7.327$); $\alpha_S = 1.488$; $\pi = 0.75$; $\gamma_S = 0.5$; $\lambda_S = 0.02$; $\delta_S = 0.012$. The lower-leftward panel shows the estimated slopes (transformed to σ values) from the mean of the β-posterior distribution. The black line represents the value of the spread of the model ($\sigma = 1$). The last red dot is the final slope estimate that corresponds to the dashed blue line. On the lower-rightward panel, the model psychometric function is represented in black and the psychometric function estimated from the staircase is represented with a dashed blue line with $\gamma = 0.5$ and $\lambda = 0.02$.

A.3.3 Psi-marginal

This Bayesian adaptive method, proposed by Prins (2013), is a flexible modification of the original Psi method that can be used to estimate the three parameters (e.g. threshold, slope, and lapse rate) of the psychometric function, or estimate one or two parameters and marginalize the non-interested parameters (i.e. nuisance parameters). For example, if we are interested in estimating the threshold (θ) and the slope (i.e. spread, σ) in a 2AFC task, we can fix the guess value to $\gamma_M = 0.5$ and marginalizing the lapse rate (in Prins' terminology Psi $\theta, \sigma(\lambda)$). The advantage of marginalizing the lapse rate, is that this method can present stimuli at particular intensities in order to obtain information about the marginalized parameter but only if those intensities reduce the uncertainty regarding the estimation of the threshold and the slope (Prins, 2013). Thus, this method can also estimate the value of the marginalized parameters although they will not be very precise. The Psi-marginal method works exactly in the same way as the original Psi method described in A.3.2. However, in order to marginalize one parameter, we have to reduce the n-dimensional posterior distribution to n-1 dimensions. This is done simply by summing across the marginalized dimension (see equation 4 of Prins, 2013). In order to implement the Psi-marginal we used the Palamedes Matlab routine *PAL_Entropy.m* (Palamedes version 1.6.3; Prins & Kingdom, 2018).

The Psi-marginal also allows the experimenter to fix the nuisance parameter (e.g. lapse rate or slope) with a known or an assumed parameter. Thus, the Psi-marginal with fixed lapse and guess rates corresponds to the original Psi method.

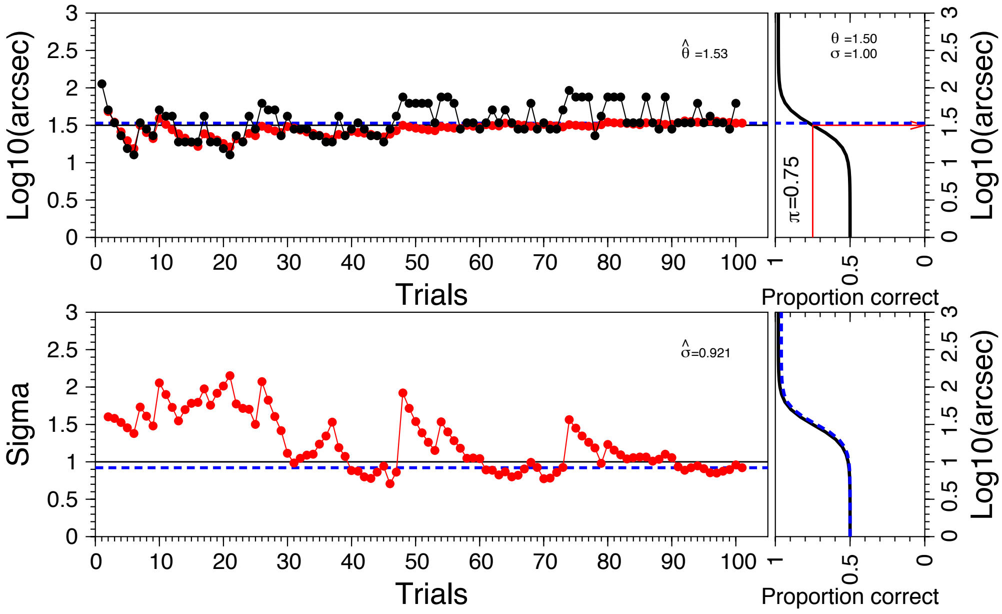

Fig. A6. Simulation example of Psi-Marginal, **Psi $\boldsymbol{\theta}, \boldsymbol{\sigma}(\boldsymbol{\lambda})$.** Same details as in Fig. A5. Note that although the lapse rate (λ) was marginalized, it can be estimated, $\hat{\lambda}$=0.039.

In the simulations presented here, we used three configurations of the Psi-marginal method. In all cases, we fixed the value of the guess rate for both tasks 2AFC ($\gamma_M = 0.5$) and 4AFC ($\gamma_M = 0.25$). One configuration is, to estimate the threshold and the slope marginalizing the lapse rates (Psi $\theta, \sigma(\lambda)$), the second configuration is to estimate the threshold and marginalizing the slope and the lapse rate (Psi $\theta, (\sigma, \lambda)$), and the third configuration is to estimate the threshold, marginalize the slope, and fix the lapse rate (Psi $\theta(\sigma)$). In the first two configurations we used a three-dimensional array composed of 50 thresholds × 50 slopes × 11 lapse rates (between 0 and 0.1 in steps of 0.01 for 2AFC, and between 0 and 0.15 in steps of 0.015 for 4AFC task). In the third configuration we fixed the lapses rate for the 2AFC task ($\gamma_M = 0.5$, $\lambda_M = 0.02$) and for the 4AFC task ($\gamma_M = 0.25$, $\lambda_M = 0.03$), so we used a two-dimensional array of 50 thresholds × 50 slopes but we marginalize the slope parameter. The range of thresholds and slopes was the same as described for the procedure ZEST 2D and Psi method (see sections A3.1 and A.3.2). The range of stimulus intensities used to calculate the expected entropy was set from $\log_{10}$(3.16 arcsec) to $\log_{10}$(1000 arcsec) in steps of 0.0862 log units (in total, 30 different stimulus disparities). An example of a simulation using the configuration Psi $\theta, \sigma(\lambda)$ can be seen in Fig. A6.

References

Alcalá-Quintana, R. y García-Pérez, M. A. (2004). The role of parametric assumptions in adaptive bayesian estimation. *Psychological Methods*, *9*, 250–271.

Anderson, A. J. (2003). Utility of a dynamic termination criterion in the ZEST adaptive threshold method. *Vision Research*, *43*, 165–170.

Emerson, P. L. (1986). Observations on maximum-likelihood and Bayesian methods of forced-choice sequential threshold estimation. *Perception & Psychophysics, 39,* 151–153.

García-Pérez, M. A. (1998). Forced-choice staircases with fixed steps sizes: Asymptotic and small-sample properties. *Vision Research*, 38, 1861–1881.

Green, D. M. (1990) Stimulus selection in adaptive psychophysical procedures. *Journal of the Acoustical Society of America*. 97 (6), 2662-2674

Green, D. M. (1993). “A maximum-likelihood method for estimating thresholds in a yes-no task,” *Journal of the Acoustical Society of America*. 93, 2096–2105.

King-Smith, P. E., Grigsby, S. S., Vingrys, A. J., Benes, S. C., & Supowit, A. (1994). Efficient and unbiased modifications of the QUEST threshold method: Theory, simulations, experimental evaluation and practical implementation. *Vision Research*, 34, 885–912.

King-Smith, P. E., & Rose, D. (1997). Principles of an Adaptive Method for Measuring the Slope of the Psychometric Function. *Vision Research*. 37 (12), 1595-1604.

Kingdom, F. A. A., & Prins, N. (2010). *Psychophysics A Practical Introduction*. London: Elsevier.

Kontsevich, L.L. & Tyler, C.W. (1999). Bayesian adaptive estimation of psychometric slope and threshold. *Vision Research*, 39, 2729-2737.

Madigan, R. & Williams, D. (1987). Maximum-likelihood psychometric procedures in two-alternative forced choice: Evaluation and Recommendations. *Perception & Psychophysics*. 42 (3), 240-249

Pelli, D. G. (1987). The ideal psychometric procedure. *Investigative Ophthalmology & Visual Science*, 28(3, Suppl.), 366.

Pentland, A. (1980). Maximum likelihood estimation: The Best PEST. *Perception & Psychophysics, 28 (4),* 377–379.

Prins, N. (2013). The psi-marginal adaptive method: how to give nuisance parameters the attention they deserve (no more, no less). *Journal of Vision*, 13(7):3, 1-17. doi: 10.1167/13.7.3

Prins, N. & Kingdom, F. A. A. (2018) Applying the Model-Comparison Approach to Test Specific Research Hypotheses in Psychophysical Research Using the Palamedes Toolbox. *Frontiers in Psychology*, 9:1250. doi: 10.3389/fpsyg.2018.01250

Serrano-Pedraza, I., Herbert, W., Villa-Laso, L., Widdall, M., Vancleef, K., & Read, J.C.A. (2016b). The stereoscopic Anisotropy Develops During Childhood. *Investigative Ophthalmology and Visual Science*. 1;57(3):960-70. doi: 10.1167/iovs.15-17766.

Shen, Y. Richards, V.M. (2012). A maximum-likelihood procedure for estimating psychometric functions: Thresholds, slopes, and lapses of attention. *J. Acoust. Soc. Am.* 132(2), 957-967.

Snoeren, P. R., & Puts, M. J. (1997). Multiple parameter estimation in an adaptive psychometric method: MUEST, an extension of the QUEST method. *Journal of Mathematical Psychology*, 41, 431–439

Taylor, M. M. (1971). On the efficiency of psychophysical measurement. *Journal of the Acoustical Society of America*. 49, 505-508.

Watson, A. B. (2017). QUEST +: A general multidimensional Bayesian adaptive psychometric method. *Journal of Vision*, 17(3):10, 1–27, doi:10.1167/17.3.10.
